# Efficient Synthesis of 2,3′-Spirobi (Indolin)-2′-Ones and Preliminary Evaluation of Their Damage to Mitochondria in HeLa Cells

**DOI:** 10.3389/fphar.2021.821518

**Published:** 2022-02-23

**Authors:** Huajie Li, Zhenjie Yu, Haoyi Sun, Bo Liu, Xin Wang, Zhe Shao, Meiling Wang, Weilin Xie, Xingang Yao, Qingqiang Yao, Ying Zhi

**Affiliations:** ^1^ School of Pharmacy and Pharmaceutical Sciences, Shandong First Medical University, Jinan, China; ^2^ Institute of Materia Medica, Shandong Academy of Medical Sciences, Jinan, China; ^3^ School of Pharmaceutical Sciences, Southern Medical University, Guangzhou, China

**Keywords:** spirooxindole, aza-ortho-quinone methides, mitochondria, morphology, annulation

## Abstract

A novel formal (4 + 1) annulation between *N*-(*o*-chloromethyl)aryl amides and 3-chlorooxindoles through *in situ* generated aza-*ortho*-QMs with 3-chlorooxindoles is reported for the synthesis of a series of 2,3′-spirobi (indolin)-2′-ones in high yields. Under structured illumination microscopy, compound **3a** is found to change the mitochondrial morphology and induce mitophagy pathway, which might then trigger mitophagy in cancer cells.

## 1 Introduction

The high prevalence and fatal incidence of cancer in the population worldwide has fueled an intensified search for new therapeutic treatment options. Chemotherapy is one of the most common strategies. The major challenging factors in developing cancer chemotherapeutics is to increase selectivity and to reduce side effects toward normal cells and tissues. (Wheeler et al., 2013) Since the efficacy and toxicity of a drug is closely associated with its subcellular distribution, interest in subcellular organelle-targeting theranostics is substantially increasing. (Kang, 2018).

Among organelles, mitochondria which is a regulatory center for cellular energy metabolism, substance synthesis and death, function as dynamic networks that often come in varied morphologies and subcellular distribution to fulfill their multiple tasks and thus have received substantial attention. (Li et al., 2020; Chen H. et al., 2021; Zou et al., 2021) Amount of researches disclosed that many human diseases have been closely related with functional mitochondria, such as neurodegenerative disorders, cardiovascular disorders, metabolic disorders, and cancers. (Cho et al., 2020) Recent studies demonstrated dramatic alterations in mitochondrial form during the early stages of cell apoptosis that is a fragmentation of the network and the remodeling of the cristae, indicating mitochondria are closely associated with apoptotic pathways. (Karbowski and Youle, 2003) Moreover, accumulating evidence indicates that the occurrence, development and metastasis of tumors has been linked to mitochondrial dysfunction and malfunctions, whose morphology is sensitive to their effects, featuring mitochondria a striking target in the design of anti-cancer drugs. (Mo et al., 2012; Hao et al., 2019) So far, some interesting and innovative examples have been reported, such as the increased anti-tumor effect of photodynamic therapy through the regulation of mitochondrial form by paclitaxel. (Zhao et al., 2017) However, these therapies are not yet in the preclinical phase. Therefore, the search for new natural or synthetic compounds that can target mitochondria as anticancer treatment is imperative.

**FIGURE 1:**
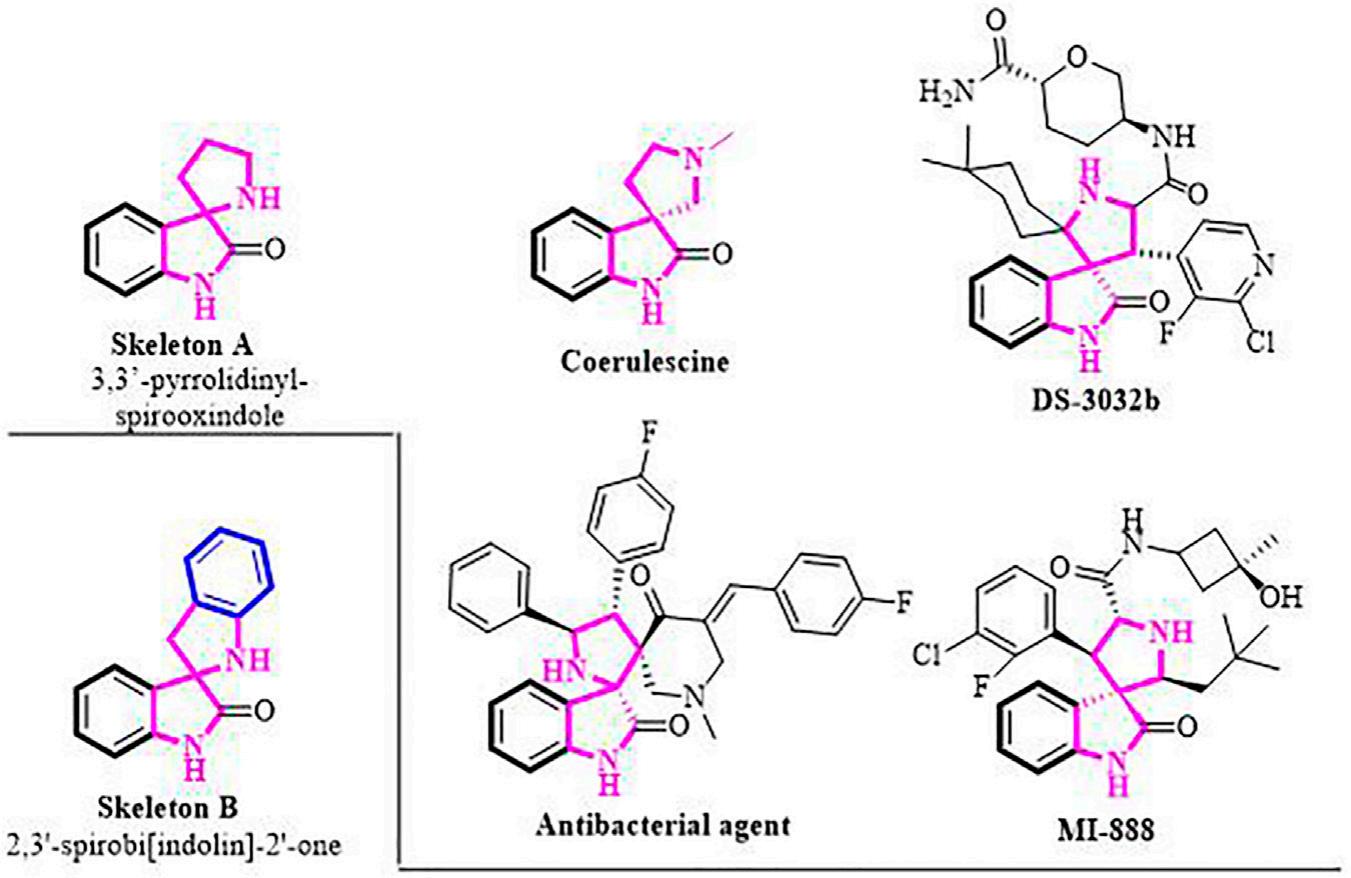
Representative biologically active 3,3′-pyrrolidinyl-spirooxindoles.

The 3,3′-pyrrolidinyl-spirooxindole skeleton is a privileged class of heterocyclic motifs, which form the core of a large family of bioactive oxindole alkaloids and medicinally important compounds. (Kumar et al., 2008; Girgis, 2009; Zhao et al., 2013a; Zhao et al., 2013b; Arumugam et al., 2021; Liu et al., 2021) For instance, coerulescine, the simplest prototype member, was isolated from *Horsfieldia superba*, extracts of which have found use in indigenous medicine. (Neil et al., 1997) Spirooxindole derivative DS-3032b exhibits MDM2 inhibitory activity employed in the treatment of patients with advanced solid tumors and lymphomas (Figure 1). (Gounder et al., 2016) Their notable biological activities prompted the development of numerous strategies toward the syntheses of 3,3′-pyrrolidinyl-spirooxindole moiety. (Cao and Zhou, 2015; He et al., 2020; Liu X. et al., 2020; Nakamura et al., 2020; Reddy et al., 2020; Bortolami et al., 2021; Nasri et al., 2021; Saranya et al., 2021) Nevertheless, the construction of the structurally similar spirobi (indolin) frameworks (Skeleton B, Figure 1 bottom left) has been less studied, and until now, only two synthetic methods have been reported for the synthesis of 2,3′-spirobi (indolin)-2′-ones. (Gui et al., 2019; Wang et al., 2019) In 2019, Shi and co-workers pioneered the (4 + 1) annulation of 3-isothiocyanato oxindoles and aza-*o*-quinone methides, affording the corresponding condensed products in two steps. Meanwhile, Zhong’s group reported an ioide salts catalyzed functionalization of carbonyl compounds with sulfonamides.

**SCHEME 1:**
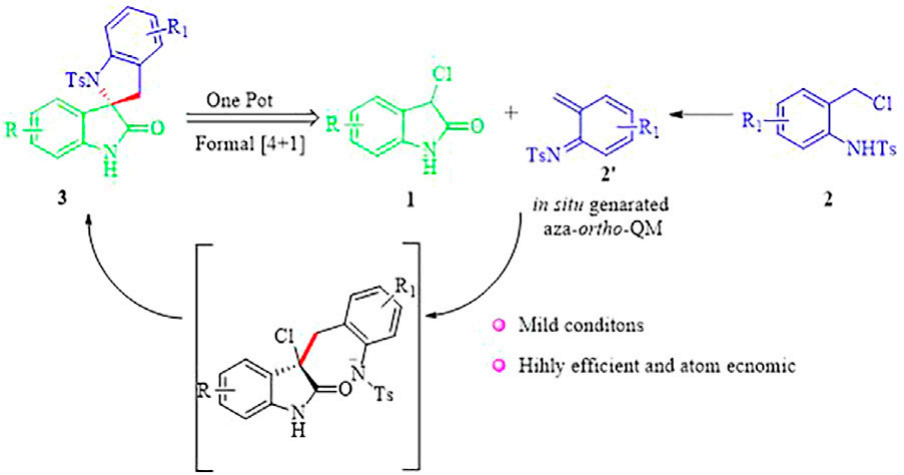
Strategy for the synthesis of 2,3′-spirobi (indolin)-2′-ones.

Although these were elegant and creative strategies, it is still highly desirable to develop a concise protocol to construct the 2,3′-spirobi (indolin)-2′-ones framework from readily available starting materials, especially under mild conditions. Based on our research expertise in the field of domino-cycloaddition, (Enders et al., 2015; Zhao et al., 2016a; Zhao et al., 2016b; Zhi et al., 2016; Zhi et al., 2018) we envisioned that the assembly of 2,3′-spirobi (indolin)-2′-ones 3 could be realized through a formal (4 + 1) reaction between *in situ* generated aza-*ortho*-QM 2′ from *N*-(*o*-chloromethyl) aryl amide 2 and 3-chloroindolin-2-one 1 in the presence of an appropriate base (Scheme 1). We hope this annulation reaction could provide a general and straightforward method to access 2,3′-spirobi (indolin)-2′-ones 3 that will serve as the basis for evaluation of bioavailabilty especially their effect on mitochondria which is understudied.

**SCHEME 2:**
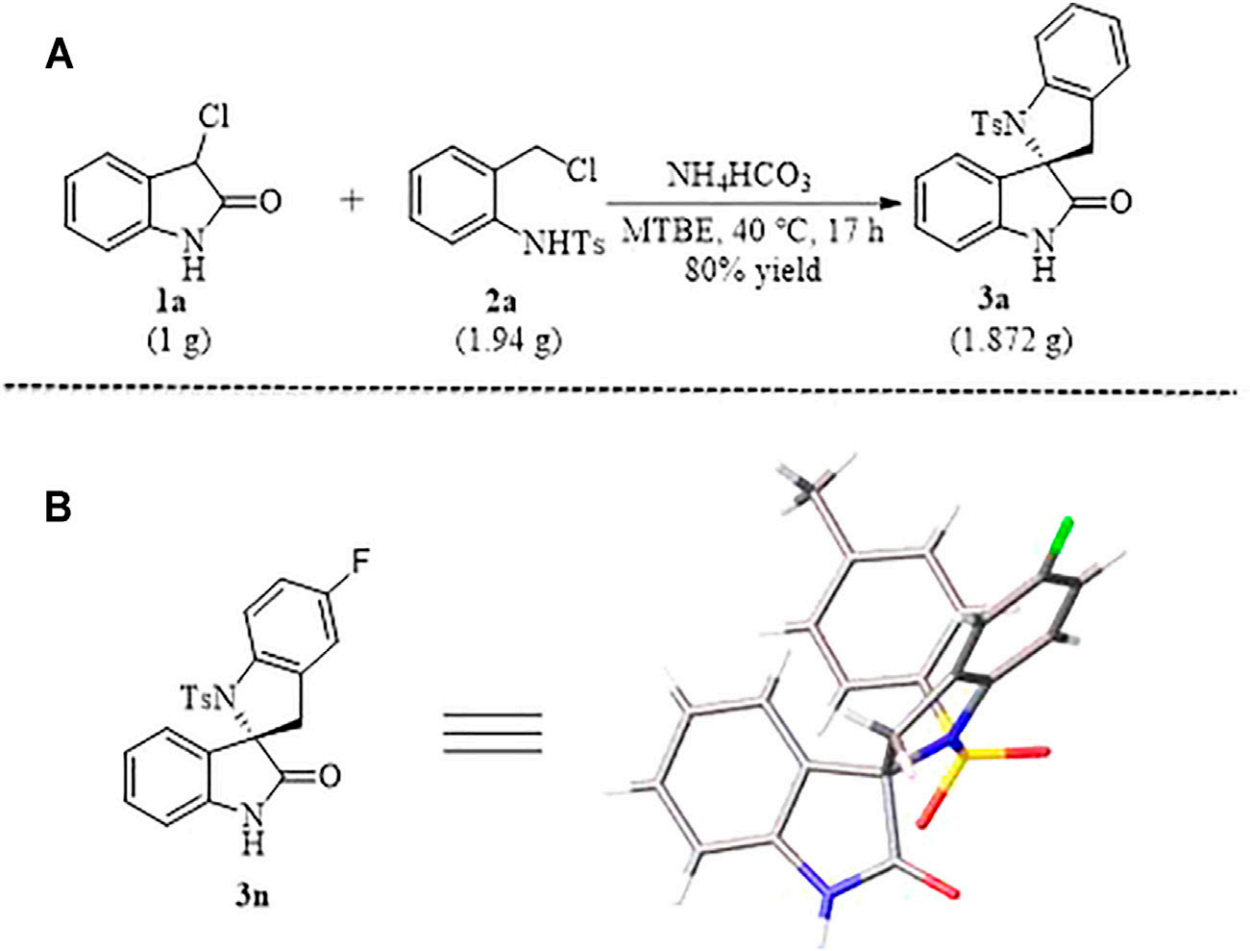
Gram-Scale synthesis **(A)** of N-tosylated spirobi (indolin) **3a** and X-ray Structure **(B)** of **3n**.

## 2 Results and Discussions

### 2.1 Chemistry

To test the feasibility of our hypothesis, we chose 3-chloroindolin-2-one **1a** and N-[2-(chloromethyl) phenyl]-4-methylbenzenesulfonamide **2a** as the model substrates to optimize the reaction conditions (Table 1). First, an initial experiment was conducted in ethyl ester at room temperature in the presence of Cs_2_CO_3_. To our delight, the expected product **3a** was obtained in a yield of 15% (Table 1, entry 1). To improve the reaction yield, the commonly used organic base Et_3_N was tested while there was no compound **3a** obtained. We found that the use of the suitable base is very crucial for the success of this reaction and thus an extensive screening of base was performed (Table 1, entries 3–6). Fortunately, inorganic bases K_2_CO_3_ and NH_4_HCO_3_ delivered the desired product **3a** in 80 and 82% yield respectively. Striving for higher efficiency, kinds of solvents and different temperature were screened and the best result was obtained by raising the reaction temperature to 40°C and using MTBE as the solvent, leading to the desired product **3a** in a yield of 88% (Table 1, entry 17).

**TABLE 1:** Reaction condition optimization studies.^a^ 
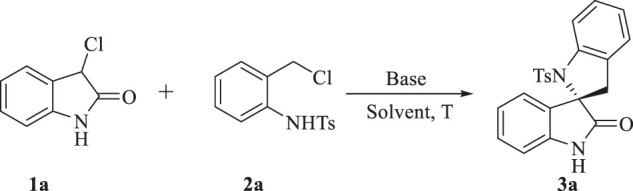

Entry	Base	Solvent	Yield^b^ (%)
1	Cs_2_CO_3_	EA	15
2	Et_3_N	EA	—
3	NaHCO_3_	EA	38
4	Na_2_CO_3_	EA	66
5	NaOH	EA	11
6	K_2_CO_3_	EA	80
7	NH_4_HCO_3_	EA	82
8	NH_4_HCO_3_	DCM	65
9	NH_4_HCO_3_	CHCl_3_	52
10	NH_4_HCO_3_	Et_2_O	61
11	NH_4_HCO_3_	Toluene	73
12	NH_4_HCO_3_	DCE	70
13	NH_4_HCO_3_	MTBE	85
14	NH_4_HCO_3_	CCl_4_	54
15^c^	NH_4_HCO_3_	MTBE	88
16^d^	NH_4_HCO_3_	MTBE	80

a All reactions were conducted with 0.4 mmol of **1a** (1.0 equiv.), 0.44 mmol of **2a** (1.1 equiv.), and 1.2 mmol of base in 4.0 ml of solvent at rt.

b Yield of isolated compound **3a** after chromatography.

c The reaction was conducted at 40°C.

d The reaction was conducted at 50°C.

All the reactions were conducted with 0.4 mmol of 1 (1.0 equiv.), 0.44 mmol of 2 (1.1 equiv.) and 1.2 mmol of base in MTBE (4.0 mL) at 40°C. Yields are those of the isolated products **3a**–**3n** after column chromatography.

Having identified the optimal reaction conditions, the substrate scope of the new protocol was explored and the results are shown in Table 2. Initially, we examined the generality of 3-chloro isatin component. A variety of isatins **1** underwent the formal (4 + 1) aunulation reaction to furnish **3b**-**3g** in 70–90% yield. Notably, substrates bearing electron-donating (R = Me, OCF_3_) or electron-withdrawing groups (R = Cl, Br) at the C5 position of the phenyl ring of **1** underwent this annulation process to furnish the corresponding products in good to excellent efficiencies **(3b-3e)**. Moreover, the C7 position substituted compounds were suitable substrates, and the target products **3f-3g** were synthesized with good results under the optimal condition. However, if fluoro group was introduced at the C7 position of isatin **1**, the yield of the reaction under the optimal condition was very low. Next, the substrate scope of this reaction was examined further by varying the reaction partner **2**. We found that all the substrates **2h**-**2n** reacted efficiently with 1a, furnishing the desired products (Table 2, **3h–3n**) in 70–92% yield. The substitution groups on the tosyl benzene ring were well tolerated and delivered the desired compounds with high efficiency (Table 2, **3h–3j**). Especially, substrates bearing electron-with-drawing groups (R_1_ = Cl, Br) on the phenyl ring of **2** readily ccould be easily processed to give the products in good to excellent yields (**3j–3n**).

**TABLE 2:** Substrate scope.
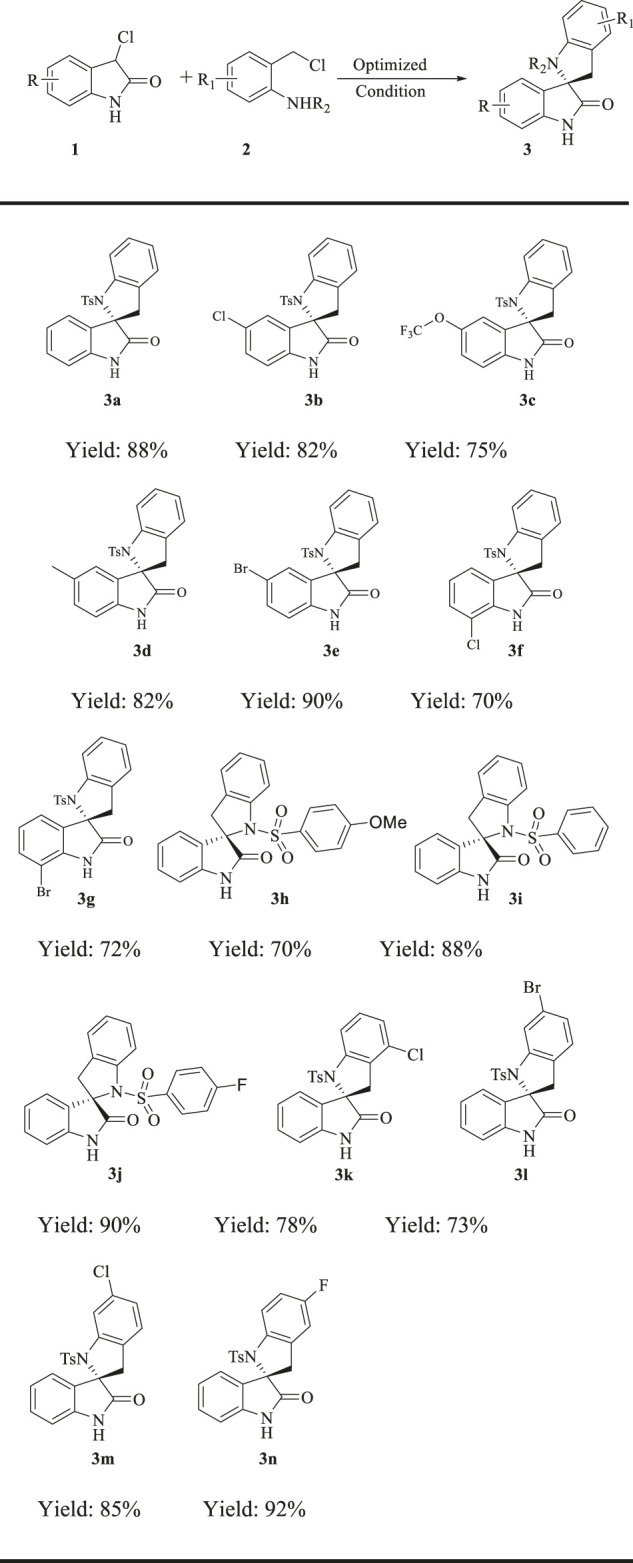

All the reactions were conducted with 0.4 mmol of 1 (1.0 equiv.), 0.44 mmol of 2 (1.1 equiv.) and 1.2 mmol of base in MTBE (4.0 mL) at 40°C. Yields are those of the isolated products 3a–3n after column chromatography.

In order to test the robustness and general utility of this 1,4-addition reaction, a gram-scale reaction was carried out under the optimal conditions and the expected product **3a** could be isolated in 80% yield without erosion of the efficiency of this process (Scheme 1A). In addition, as shown in Scheme 1B, the relative configuration of compound **3n** was determined unambiguously by X-ray crystallography.

### 2.2 Super-resolution Imaging Reveals **3a (LHJ-090)** Changes Mitochondrial Morphology and Distribution

**FIGURE 2:**
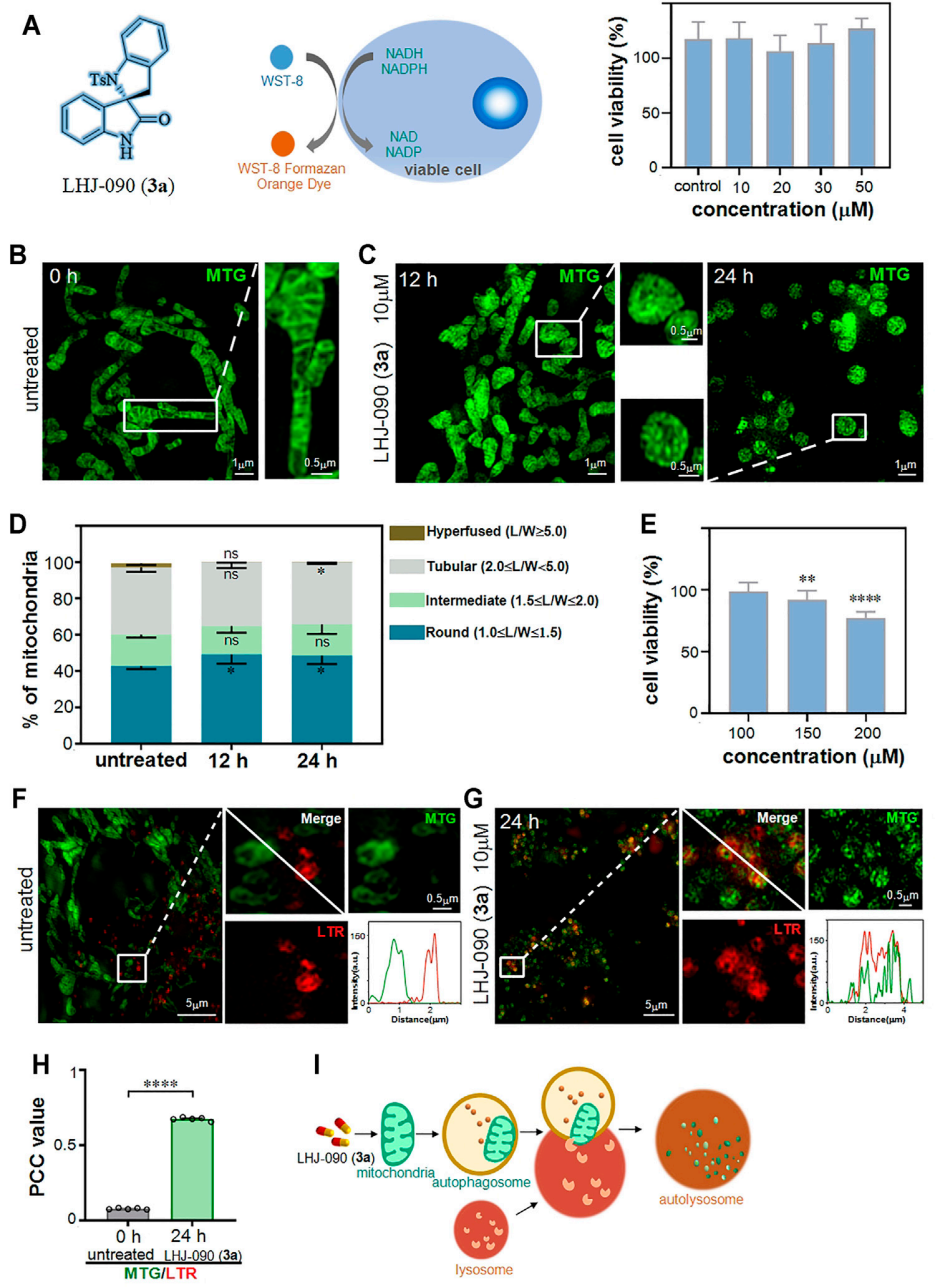
LHJ-090 (**3a**) damage mitochondria and stimulate the process of mitophagy. **(A)** The cell viability (%) obtained with cck8 assay. Percentage of viable HeLa cells after treated with different concentrations of **3a** (0 10, 20, 30 and 50 μM) for 24 h **(B,C)** SIM imaging of mitochondria in HeLa cells were treated with **3a** (10 μM) for 0, 12, and 24 h and then stained with the mitochondrial tracker probe (mito-tracker-green, MTG) (λex = 488 nm) for 0.5 h. **(D)** Quantitative analyze of mitochondrial morphology in HeLa cells after treated with **3a** for 0, 12, and 24 h. Data was appeared as Mean ± SEM (*n* = 5). *p < 0.05, all compared with untreated cells. **(E)** The cell viability (%) obtained with CCK-8 assay at high concentration **3a** stimulation, more than 150 μM shows toxicity to cells. **(F,G)** SIM colocalization images of MTG-stained mitochondria and LTR-stained lysosome with **(G)** or without **(F) 3a** treatment, the white solid square indicates fluorescence intensity. **(H)** The *PCC* values for MTG and LTR in HeLa cells from **(F)** and **(G)**. **(I)** A schematic diagram of the role of **3a** in mitochondrial damage.

After the series of 2,3′-spirobi (indolin)-2′-ones were synthesized, **3a** was selected to evaluate its damage effect on mitochondria. To verify the cytotoxicity of **3a**, we chose a colorimetric measurement tool commonly used in laboratories, CCK-8, (Lou et al., 2010) which relies on WST-8 that can be reduced by mitochondrial dehydrogenase (such as succinate dehydrogenase, SDH) to produce a highly water-soluble orange-yellow formazan product for counting the number of live cells (Figure 2A). (Liu L. Y. et al., 2020) We found that the cells did not respond to the detection threshold for the CCK-8 assay after treatment with **3a** at the concentration ranging from 10 to 50 μM. As it is generally accepted that the activity of SDH was applied as an indicator to evaluate the tricarboxylic acid cycle for reflecting cell activity rather than mitochondria behavior, (Farshbaf and Kiani-Esfahani, 2018) colorimetric tools based on a large number of cells are inaccurately for clarifying the regulation of drugs on a single mitochondria.

To more accurately reflect the damage of **3a** to the mitochondria, we applied recently developed structured illumination microscopy (SIM), a new tool for investing the effect of drugs at the single mitochondria level in living cells. (Wei et al., 2022) SIM based on a known spatially structured pattern of light to excite a sample whose fringe position and direction can be changed multiple times and to record the emission fluorescence signal at each position, thereby providing up to 100 nm spatial resolution. (Chen et al., 2018) Therefore, this tool can help us accurately and quantitatively study the behavior of **3a** at the nanoscale in living cells system.

Next, we checked the **3a** at the concentration of 10 μM in HeLa cells, and then observed it under SIM. We used a commercial mitochondrial probe (Mito-Tracker Green, MTG) to label mitochondria in HeLa cells after **3a** stimulation. (Chen et al., 2020a; Zhang et al., 2021) Compared to the SIM images captured at 0 h (Figure 2B), we observed that mitochondrial morphology has changed from fibrous-like to round-like after the **3a** treatment for 12 and 24 h (Figure 2C), showing the mitochondria were destroyed. To evaluate the mitochondrial morphology, the length-to-width ratio (*L*/*W*), was introduced as previously reported. (Shao et al., 2020) This system propose four standards to measure the morphology of mitochondria, namely round or nearly round (1.0 ≤ *L*/*W* < 1.5), intermediate (1.5 ≤ *L*/*W* < 2.0), tubular (2.0 ≤ *L*/*W* < 5.0), and hyperfused (*L*/*W* ≥ 5.0). We then quantify the distribution of individual mitochondria in HeLa cells, and found that the distribution of mitochondrial morphology was changed with the **3a** treatment for 12 and 24 h (Figure 2D), indicating that **3a** at 10 μM could damage mitochondrial morphology’s distribution.

Finally, we increased the concentration of **3a** to check the detection threshold of CCK-8, and found that it could not be responded until 150 μM (Figure 2E), which shows that SIM is more accurate in exploring the sensitivity of drugs to subcellular behavior.

### 2.3 **3a** Damages Mitochondria Which Then Involved in the Process of Mitophagy

Mitophagy is a process by which cells remove and degrade damaged mitochondria, and its typical feature is the overlap of lysosomes and mitochondria. (Chen et al., 2020b; Chen Q. et al., 2021) After clarifying that **3a** can damage mitochondria, we further studied whether drug-induced mitochondrial damage is involved in the mitophagy pathway.

We then use MTG and commercial lysosomal probe (Lyso-Tracker Red, LTR) to simultaneously label drug-treated HeLa cells. (Wang et al., 2020; Zhang C. et al., 2021) Results revealed that the mitochondria was damaged to be granular after 24 h of the drug treatment as the green mitochondria stained by MTG and the red lysosome stained by LTR overlapped into yellow (Figure 2F). Compared with that in untreated cells, the overlap of mitochondria and lysosome in cells treated with **3a** for 24 h was increased significantly (Figure 2G). Together, these results suggested that **3a** induced the change of mitochondrial morphology, and then triggered the mitophagy pathway.

## 3 Conclusion

Taken together, we reported a novel (4 + 1) annulation reaction between 3-chlorooxindoles and *N*-(*o*-chloromethyl) aryl amides through *in situ* generated aza-*ortho*-QM with 3-chlorooxindoles for the efficient synthesis of various 2,3′-spirobi (indolin)-2′-ones in good to excellent yield under mild conditions. By using the highly accurate tool structured illumination microscopy, we found that compound 3a could damage the distribution of mitochondrial form and induce mitophagy pathway, which finally might promote the mitophagy in cancer cells. Further efforts are in progress to evaluate the antiproliferative activity of these spiropyrrolidine analogs against tumor cell lines.

## 4 Experimental Sections

### 4.1 Chemistry

#### 4.1.1 General Information

The chemical reagents are commercially available and were used without further purification. Reactions were monitored by Thin Layer Chromatography (TLC) (Silica gel HF254 or GF254 from Qingdao Haiyang Chemical Co., Ltd., Qingdao, China), and the spots were visualized with ultraviolet irradiation (254 nm). Compounds were purified by solvent beating or silica gel column chromatography (200–300 mesh). ^1^H NMR and ^13^C NMR spectra were recorded on a Bruker AVANCE AV III 600 spectrometer using CDCl_3_ or d-DMSO as solvent. Data for ^1^H NMR are reported as follows: chemical shift (ppm), multiplicity (s = singlet, d = doublet, t = triplet, q = quartet, dd = doublet of doublet, td = triplet of doublet, m = multiplet, br = broad), integration, and coupling constant (Hz). Data for ^13^C NMR are reported in terms of chemical shift and multiplicity where appropriate. High resolution mass spectra (HRMS) were obtained from Thermo Scientific Q Exactive Plus. The melting points were determined by Büchi 510 apparatus without corrected.

#### 4.1.2 General Procedure for the Synthesis of Products **3a**-**3n**

To an oven-dried flask were added **1** (0.4 mmol, 66.8 mg, 1.0 equiv), **2** (0.44 mmol, 130 mg, 1.1 equiv) and NH_4_HCO_3_ (1.2 mmol, 94.9 mg, 3.0 equiv) followed by the addition of MTBE (4.0 ml). The reaction mixture was allowed to stir at 40°C for 17 h and then directly poured into water. The solution was extracted with dichloromethane (3 × 15 ml). The organic phases were combined, washed with brine and dried over Na_2_SO_4_. Then the solvent was evaporated to give a crude product which was purified by silica gel chromatography (hexane/ethyl acetate = 10/1 to 4/1) to provide the desired products **3a**-**3n**. The scale-up synthesis of **3a** was the same as the above steps.

(S)-1-Tosyl-2,3′-spirobi (indolin)-2′-one (3a)

According to general procedure, the crude product was purified by silica gel chromatography (hexane/ethyl acetate = 10/1 to 4/1) to provide **3a** as a white solid (132.6 mg, 88% yield). mp: 267–269°C.^1^H NMR (400 MHz, DMSO-*d*_6_) *δ* 10.76 (s, 1H), 7.70 (d, *J* = 8.4 Hz, 2H), 7.33 (d, *J* = 8.4 Hz, 2H), 7.28–7.17 (m, 4H), 7.04–7.00 (m, 1H), 6.94 (d, *J* = 8.4 Hz, 1H), 6.83 (d, *J* = 7.6 Hz, 1H), 6.76 (d, *J* = 7.6 Hz, 1H), 3.54 (d, *J* = 16.0 Hz, 1H), 3.23 (d, *J* = 16.4 Hz, 1H), 2.36 (s, 3H) ppm; ^13^C NMR (150 MHz, CDCl_3_) *δ* 177.5, 144.1, 141.6, 139.5, 136.4, 130.9, 129.8, 129.5 (2C), 128.1, 128.0 (2C), 127.2, 125.2, 123.0, 123.0, 122.9, 112.5, 110.6, 71.7, 42.2, 21.6 ppm. HRMS (ESI): m/z (M + H)^+^ calcd for C_22_H_19_N_2_O_3_S^+^ 391.1116; found 391.1112.

(S)-5′-Chloro-1-Tosyl-2,3′-Spirobi (indolin)-2′-One (3b)

According to general procedure, the crude product was purified by silica gel chromatography (hexane/ethyl acetate = 10/1 to 4/1) to provide **3b** as a white solid (140 mg, 82% yield). mp: 253–255°C. ^1^H NMR (400 MHz, DMSO-*d*_6_) *δ* 10.91 (s, 1H), 7.59 (d, *J* = 8.0 Hz, 2H), 7.36–7.23 (m, 6H), 7.05 (t, *J* = 7.2 Hz, 1H), 6.95 (d, *J* = 8.4 Hz, 1H), 6.50 (s, 1H), 3.51 (d, *J* = 16.4 Hz, 1H), 3.30 (d, *J* = 16.4 Hz, 1H), 2.36 (s, 3H) ppm; ^13^C NMR (100 MHz, DMSO-d6) *δ* 176.8, 145.0, 141.5, 140.6, 136.3, 131.7, 130.1 (2C), 130.0, 128.6, 127.8, 127.6, 126.3, 126.0, 123.6 (2C), 123.3, 112.5, 112.4, 71.4, 41.7, 21.5 ppm; HRMS (ESI): m/z (M + H)^+^ calcd for C_22_H_18_ClN_2_O_3_S^+^ 425.0727; found 425.0718.

(S)-1-Tosyl-5'-(trifluoromethoxy)-2,3′-spirobi (indolin)-2′-one (3c)

According to general procedure, the crude product was purified by silica gel chromatography (hexane/ethyl acetate = 10/1 to 4/1) to provide **3c** as a white solid (142 mg, 75% yield). mp: 98–102°C. ^1^H NMR (600 MHz, DMSO-*d*_6_) *δ* 10.96 (s, 1H), 7.69 (d, *J* = 8.4 Hz, 2H), 7.34 (d, *J* = 8.4 Hz, 2H), 7.30 (d, *J* = 8.4 Hz, 1H), 7.27 (d, *J* = 7.8 Hz, 1H), 7.24–7.20 (m, 2H), 7.05–7.02 (m, 2H), 6.74 (s, 1H), 3.54 (d, *J* = 16.2 Hz, 1H), 3.33 (d, *J* = 15.6 Hz, 1H), 2.36 (s, 3H) ppm; ^13^C NMR (150 MHz, DMSO-*d*_6_) *δ* 177.1, 145.0, 143.6, 143.5 141.5, 140.9, 136.4, 132.1, 130.2 (2C),128.5, 127.7 (2C), 125.9, 123.6, 116.8, 112.4, 111.8, 71.7, 41.8, 21.4 ppm; HRMS (ESI): m/z (M + H)^+^ calcd for C_23_H_18_F_3_N_2_O_4_S^+^ 475.0939; found 475.0935.

(S)-5′-Methyl-1-Tosyl-2,3′-Spirobi (indolin)-2′-One (3d)

According to general procedure, the crude product was purified by silica gel chromatography (hexane/ethyl acetate = 10/1 to 4/1) to provide **3d** as a white solid (132 mg, 82% yield). mp: 282–284°C. ^1^H NMR (400 MHz, DMSO-*d*_6_) *δ* 10.64 (s, 1H), 7.55 (d, *J* = 8.0 Hz, 2H), 7.34–7.25 (m, 5H), 7.07–7.02 (m, 2H), 6.83 (d, *J* = 8.0 Hz, 1H), 6.34 (s, 1H), 3.51 (d, *J* = 16.4 Hz, 1H), 3.20 (d, *J* = 18.8 Hz, 1H), 2.36 (s, 3H), 2.01 (s, 3H) ppm; ^13^C NMR (150 MHz, CDCl_3_) *δ* 177.5, 143.9, 141.8, 137.1, 136.6, 132.5,130.4, 130.1, 129.3 (2C), 128.1, 127.9 (2C), 127.3, 125.2, 123.7, 123.0, 112.6, 110.3, 71.6, 42.2, 21.5, 20.8 ppm; HRMS (ESI): m/z (M + H)^+^ calcd for C_23_H_21_N_2_O_3_S^+^ 405.1273; found 405.1261.

(S)-5′-Bromo-1-Tosyl-2,3′-Spirobi (indolin)-2′-One (3e)

According to general procedure, the crude product was purified by silica gel chromatography (hexane/ethyl acetate = 10/1 to 4/1) to provide **3e** as a white solid (169 mg, 90% yield). mp: 269–273°C.^1^H NMR (400 MHz, DMSO-*d*_6_) *δ* 10.91 (s, 1H), 7.56 (d, *J* = 8.0 Hz, 2H), 7.44 (d, *J* = 8.0 Hz, 1H), 7.37 (d, *J* = 8.0 Hz, 1H), 7.31–7.25 (m, 4H), 7.06 (t, *J* = 7.6 Hz, 1H), 6.91 (d, *J* = 8.0 Hz, 1H), 6.57 (s, 1H), 3.51 (d, *J* = 16.0 Hz, 1H), 3.30 (d, *J* = 16.4 Hz, 1H), 2.37 (s, 3H) ppm; ^13^C NMR (100 MHz, DMSO-*d*_6_) *δ* 176.6, 145.0, 141.5, 141.0, 136.3, 132.9, 132.0, 130.2 (2C), 128.6, 127.8, 127.5 (2C), 126.0, 125.9, 123.7, 114.0, 112.9, 112.5, 71.3, 41.7, 21.6 ppm; HRMS (ESI): m/z (M + H)^+^ calcd for C_22_H_18_BrN_2_O_3_S^+^ 469.0222; found 469.0196.

(S)-7′-Chloro-1-Tosyl-2,3′-Spirobi (indolin)-2′-One (3f)

According to general procedure, the crude product was purified by silica gel chromatography (hexane/ethyl acetate = 10/1 to 4/1) to provide **3f** as a yellow solid (119 mg, 70% yield). mp: 250–255°C.^1^H NMR (600 MHz, DMSO-*d*_6_) *δ* 11.24 (s, 1H), 7.74 (d, *J* = 8.4 Hz, 2H), 7.37 (d, *J* = 7.8 Hz, 3H), 7.26 (d, *J* = 7.2 Hz, 1H), 7.22 (t, *J* = 7.8 Hz, 1H), 7.16 (d, *J* = 8.4 Hz, 1H), 7.03 (t, *J* = 7.2 Hz, 1H), 6.88 (t, *J* = 7.8 Hz, 1H), 6.78 (d, *J* = 7.8 Hz, 1H), 3.55 (d, *J* = 16.2 Hz, 1H), 3.30 (d, *J* = 16.2 Hz, 1H), 2.37 (s, 3H) ppm; ^13^C NMR (150 MHz, DMSO-*d*_6_) *δ* 176.7, 144.5, 140.9, 138.8, 135.8, 132.3, 129.8 (2C),128.0, 127.5 (2C),127.3, 125.5, 123.5, 123.1, 121.1, 114.5, 111.8, 71.8, 41.6, 21.0 ppm; HRMS (ESI): m/z (M + H)^+^ calcd for C_22_H_18_ClN_2_O_3_S^+^ 425.0727; found 425.0725.

(S)-7′-bromo-1-tosyl-2,3′-spirobi (indolin)-2′-one (3g).

According to general procedure, the crude product was purified by silica gel chromatography (hexane/ethyl acetate = 10/1 to 4/1) to provide **3g** as a yellow solid (135 mg, 72% yield). mp: 270–274°C.^1^H NMR (400 MHz, CDCl_3_) *δ* 7.88 (d, *J* = 8.4 Hz, 2H), 7.74 (s, 1H), 7.40 (d, *J* = 8.0 Hz, 1H), 7.25 (d, *J* = 9.6 Hz, 2H), 7.21–7.15 (m, 3H), 7.01–6.93 (m, 2H), 6.80 (t, *J* = 7.6 Hz, 1H), 3.73 (d, *J* = 15.6 Hz, 1H), 3.21 (d, *J* = 15.6 Hz, 1H), 2.39 (s, 3H) ppm; ^13^C NMR (150 MHz, CDCl_3_) *δ* 176.2, 144.4, 141.4, 138.7, 136. 2, 132.5, 132.4, 129.6 (2C), 128.2, 128.1 (2C), 126.8, 125.2, 124.4, 123.1, 121.5, 112.4, 103.6, 72.9, 42.3, 21.6 ppm; HRMS (ESI): m/z (M + H)^+^ calcd for C_22_H_18_BrN_2_O_3_S^+^ 469.0222; found 469.0222.

(S)-1-[(4-methoxyphenyl)sulfonyl]-2,3′-spirobi (indolin)-2′-one (3h)

According to general procedure, the crude product was purified by silica gel chromatography (hexane/ethyl acetate = 10/1 to 4/1) to provide **3h** as a white solid (113.7 mg, 70% yield). mp: 245–248°C.^1^H NMR (600 MHz, CDCl_3_) *δ* 7.87 (d, J = 9.0 Hz, 2H), 7.71 (s, 1H), 7.25–7.23 (m, 2H), 7.19–7.15 (m, 2H), 6.99 (t, J = 7.2 Hz, 1H), 6.95 (d, J = 7.2 Hz, 1H), 6.92 (d, J = 7.8 Hz, 1H), 6.89- 6.86 (m, 3H), 3.83 (s, 3H), 3.74 (d, J = 15.6 Hz, 1H), 3.22 (d, J = 16.2 Hz, 1H) ppm; ^13^C NMR (150 MHz, CDCl_3_) *δ* 177.4, 163.3, 141.7, 139.4, 131.0, 130.9, 130.3 (2C), 129.7, 128.1, 127.2, 125.2, 123.1, 122.9, 122.9, 114.1 (2C), 112.4, 110.5, 71.7, 55.6, 42.2 ppm; HRMS (ESI): m/z (M + H)^+^ calcd for C_22_H_19_N_2_O_4_S^+^ 407.1065; found 407.1062.

(S)-1-(phenylsulfonyl)-2,3′-spirobi (indolin)-2′-one (3i)

According to general procedure, the crude product was purified by silica gel chromatography (hexane/ethyl acetate = 10/1 to 4/1) to provide **3i** as a red solid (132.5 mg, 88% yield). mp: 138–140°C.^1^H NMR (400 MHz, DMSO-*d*_6_) *δ* 10.77 (s, 1H), 7.81 (d, *J* = 7.6 Hz, 2H), 7.67 (t, *J* = 7.2 Hz, 1H), 7.53 (t, *J* = 7.6 Hz, 2H), 7.29–7.23 (m, 4H), 7.06–7.02 (m, 1H), 6.95 (d, *J* = 8.0 Hz, 1H), 6.80 (t, *J* = 7.2 Hz, 1H), 6.73 (t, *J* = 7.2 Hz, 1H), 3.55 (d, *J* = 16.2.4 Hz, 1H), 3.25 (d, *J* = 16.4 Hz, 1H) ppm; ^13^C NMR (100 MHz, DMSO-*d*_6_) *δ* 177.1, 141.6, 141.5, 139.34, 134.2, 130.8, 130.2, 129.7 (2C), 128.4, 128.0, 127.8 (2C), 125.9, 123.5, 123.0, 122.5, 112.3, 110.8, 71.8, 42.1 ppm; HRMS (ESI): m/z (M + H)^+^ calcd for C_21_H_17_N_2_O_3_S^+^ 377.0960; found 377.0955.

(S) -1-[(4-fluorophenyl)sulfonyl]-2,3′-spirobi (indolin)-2′-one (3j)

According to general procedure, the crude product was purified by silica gel chromatography (hexane/ethyl acetate = 10/1 to 4/1) to provide **3j** as a red solid (141 mg, 90% yield). mp: 207–210°C. ^1^H NMR (400 MHz, DMSO-*d*_6_) *δ* 10.80 (s, 1H), 7.88–7.85 (m, 2H), 7.38 (t, *J* = 13.2 Hz, 2H) 7.29–7.25 (m, 4H), 7.07–7.03 (m, 1H), 6.95 (d, *J* = 7.6 Hz, 1H), 6.81 (t, *J* = 7.6 Hz, 1H), 6.73 (d, *J* = 7.2 Hz, 1H) 3.55 (d, *J* = 16.4 Hz, 1H), 3.25 (d, *J* = 16.4 Hz, 1H) ppm; ^13^C NMR (125 MHz, CDCl_3_) *δ* 177.7, 141.4, 139.7, 136.4, 130.8, 130.8, 130.5, 130.0, 128.2, 127.3, 125.4, 123.3, 123.0, 122.8, 116.2, 116.1, 112.5, 110.9, 71.8, 42.2, 29.7 ppm; HRMS (ESI): m/z (M + H)^+^ calcd for C_21_H_16_FN_2_O_3_S^+^ 395.0866; found 395.0863.

(S)-4-Chloro-1-tosyl-2,3′-spirobi (indolin)-2′-one (3k)

According to general procedure, the crude product was purified by silica gel chromatography (hexane/ethyl acetate = 10/1 to 4/1) to provide **3k** as a red solid (132.6 mg, 78% yield). mp: 222–226°C.^1^H NMR (600 MHz, CDCl_3_) *δ* 8.07 (s, 1H), 7.76 (d, *J* = 7.6 Hz, 2H), 7.26 (t, *J* = 9.6 Hz, 1H), 7.22–7.18 (m, 3H), 7.14 (t, *J* = 8.4 Hz, 1H), 6.98 (d, *J* = 7.8 Hz, 1H), 6.94 (t, *J* = 7.8 Hz, 2H),6.88 (t, *J* = 7.8 Hz, 1H) 3.72 (d, *J* = 16.8 Hz, 1H), 3.30 (d, *J* = 16.2 Hz, 1H), 2.38 (s, 3H) ppm; ^13^C NMR (150 MHz, CDCl_3_) *δ* 177.1, 144.5, 142.9, 139.6, 136.2, 131.0, 130.6, 123.0, 129.7, 129.6 (2C),128.0 (2C),125.7, 123.1, 123.2, 123.0, 110.7, 110.6, 71.4, 41.5, 21.6 ppm; HRMS (ESI): m/z (M + H)^+^ calcd for C_22_H_18_ClN_2_O_3_S^+^ 425.0727; found 425.0718.

(S)-6-Bromo-1-tosyl-2,3′-spirobi (indolin)-2′-one (3l)

According to general procedure, the crude product was purified by silica gel chromatography (hexane/ethyl acetate = 10/1 to 4/1) to provide **3l** as a white solid (137 mg, 73% yield). mp: 89–93°C.^1^H NMR (600 MHz, CDCl_3_) *δ* 7.76 (d, *J* = 8.4 Hz, 2H), 7.72 (s, 1H), 7.44 (s, 1H), 7.28-7.27 (m, 1H), 7.23 (d, *J* = 8.4 Hz, 2H), 7.13–7.12 (m, 1H), 7.01 (d, *J* = 7.8 Hz, 1H), 6.93–6.90 (M, 2H), 6.87 (d, *J* = 8.4 Hz, 1H), 3.65 (d, *J* = 16.2 Hz, 1H), 3.15 (d, *J* = 15.6 Hz, 1H), 2.39 (s, 3H) ppm; ^13^C NMR (150 MHz, CDCl_3_) *δ* 176.8, 144.5, 143.0, 139.4, 136.0, 130.4, 130.0, 129.7 (2C), 128.0 (2C),126.3, 126.2, 125.9, 123.2, 123.0, 121.7, 115.7, 110.6, 72.1, 41.7, 21.6 ppm; HRMS (ESI): m/z (M + H)^+^ calcd for C_22_H_18_BrN_2_O_3_S^+^ 469.0222; found 469.0108.

(S)-6-Chloro-1-tosyl-2,3′-spirobi (indolin)-2′-one (3m)

According to general procedure A, the crude product was purified by silica gel chromatography (hexane/ethyl acetate = 10/1 to 4/1) to provide **3m** as a white solid (144.5 mg, 85% yield). mp: 174–177°C. ^1^H NMR (400 MHz, DMSO-*d*_6_) *δ* 10.79 (s, 1H), 7.69 (d, *J* = 8.0 Hz, 2H), 7.37 (d, *J* = 8.0 Hz, 2H), 7.28 (d, *J* = 7.6 Hz, 2H), 7.10 (d, *J* = 10.4 Hz, 2H), 6.94 (d, *J* = 8.0 Hz, 1H), 6.86–6.82 (m, 2H), 3.51 (d, *J* = 16.4 Hz, 1H), 3.25 (d, *J* = 16.4 Hz, 1H), 2.38 (s, 3H) ppm; ^13^C NMR (100 MHz, DMSO-*d*_6_) *δ* 176.7, 145.2, 143.0, 141.6, 136.1, 132.6, 130.4, 130.3 (2C), 127.9 (2C), 127.3, 127.2 123.2, 122.6, 112.0, 110.9, 72.6, 67.5, 41.4, 25.6, 21.5 ppm; HRMS (ESI): m/z (M + H)^+^ calcd for C_22_H_18_ClN_2_O_3_S^+^ 425.0727; found 425.0725.

(S)-5-Fluoro-1-tosyl-2,3′-spirobi (indolin)-2′-one (3n)

According to general procedure, the crude product was purified by silica gel chromatography (hexane/ethyl acetate = 10/1 to 4/1) to provide **3n** as a white solid (150 mg, 92% yield). mp: 195–198°C.^1^H NMR (400 MHz, DMSO-*d*_6_) *δ* 10.77 (s, 1H), 7.67 (d, *J* = 8.0 Hz, 2H), 7.33 (d, *J* = 8.4 Hz, 2H), 7.28 (t, *J* = 7.6 Hz, 1H), 7.19–7.16 (m, 2H), 7.05 (d, *J* = 9.2 Hz, 1H), 6.94 (d, *J* = 8.0 Hz, 1H), 6.85-6.77 (m, 2H), 3.53 (d, *J* = 15.2 Hz, 1H), 3.24 (d, *J* = 16.4 Hz, 1H), 2.37 (s, 3H) ppm; ^13^C NMR (100 MHz, DMSO-*d*_6_) *δ* 176.8, 144.9, 141.5, 138.0, 136.3, 130.6, 130.1 (2C), 127.9 (2C), 123.1, 122.5, 114.8, 114.5, 113.6, 113.3, 113.0, 112.9, 110.8, 41.8, 25.6, 21.5 ppm; HRMS (ESI): m/z (M + H)^+^ calcd for C_22_H_18_FN_2_O_3_S^+^ 409.1022; found 409.1010.

### 4.2 Biological Part

#### 4.2.1 Cell Culture

HeLa cells were cultured in Dulbecco’s modified Eagle’s medium (#11965118, DMEM, Thermo Fisher Scientific) supplemented with 10% Certified fetal bovine serum (#C04001-500, FBS, VivaCell, Shanghai, China) penicillin (100 units/ml), and streptomycin (100 μg/ml; #15140163, 10,000 units/ml, Thermo Fisher Scientific) in a 5% CO_2_ humidified incubator at 37°C.

#### 4.2.2 OMX-SIM Super Resolution Imaging

HeLa cells were incubated with MTG and LTR at 37°C for 30 min in fresh DMEM and then washed three times with fresh DMEM. Super-resolution images were acquired on a commercial OMX-3D-SIM Microscope. Images were obtained at 512 × 512 using Z-stacks with a step size of 0.125 μm. The laser model was set to fast 272 MHz, the gain was set to 1, the output powers at the fiber end: 65 mW. All fluorescence images were analyzed, and their backgrounds were subtracted with Image J software.

#### 4.2.3 Data Analysis

Statistical analysis was performed with Prism 8 (GraphPad). Normality and lognormality test to check the normal distribution. In the case of normal distribution, the statistical comparison of results was test with a Student’s t test. In the case of non-normal distribution, the statistical comparison of results was test with a Mann-Whitney test, with levels of significance set at n. s. (no significant difference), *p < 0.05, **p < 0.01, ***p < 0.001, and ****p < 0.0001. Data are presented as mean ± SEM. Analyzed cells were obtained from three replicates. Statistical significances and sample sizes in all graphs are indicated in the corresponding figure legends.

## Data Availability

The original contributions presented in the study are included in the article/Supplementary Material, further inquiries can be directed to the corresponding authors.
