# Supplementary material for: Efficient Synthesis of 2,3′-Spirobi (Indolin)-2′-Ones and Preliminary Evaluation of Their Damage to Mitochondria in HeLa Cells
Source: Front Pharmacol. 2022 Feb 23;12:821518. doi: 10.3389/fphar.2021.821518 (PMC8904893; doi:10.3389/fphar.2021.821518)

checkCIF/PLATON report

Structure factors have been supplied for datablock(s) a_sq

THIS REPORT IS FOR GUIDANCE ONLY. IF USED AS PART OF A REVIEW PROCEDURE FOR PUBLICATION, IT SHOULD NOT REPLACE THE EXPERTISE OF AN EXPERIENCED CRYSTALLOGRAPHIC REFEREE.

No syntax errors found.   CIF dictionary   Interpreting this report

Datablock: a_sq

Bond precision: C-C = 0.0026 A   Wavelength=1.34139

Cell:	a=14.4909(7)	b=10.3302(5)	c=15.6218(7)
	alpha=90	beta=96.877(2)	gamma=90
Temperature:	193 K		

	Calculated	Reported
Volume	2321.66(19)	2321.66(19)
Space group	P 21/c	P 1 21/c 1
Hall group	-P 2ybc	-P 2ybc
Moiety formula	C22 H17 F N2 O3 S [+ solvent]	C22 H17 F N2 O3 S
Sum formula	C22 H17 F N2 O3 S [+ solvent]	C22 H17 F N2 O3 S
Mr	408.44	408.43
Dx,g cm-3	1.168	1.169
Z	4	4
Mu (mm-1)	0.978	0.978
F000	848.0	848.0
F000’	851.15	
h,k,lmax	18,13,20	18,13,19
Nref	5289	5115
Tmin,Tmax	0.889,0.907	0.635,0.752
Tmin’	0.889	

Correction method= # Reported T Limits: Tmin=0.635 Tmax=0.752
AbsCorr = MULTI-SCAN

Data completeness= 0.967   Theta(max)= 60.329

R(reflections)= 0.0430(3490)   wR2(reflections)= 0.1134(5115)

S = 1.049   Npar= 263

The following ALERTS were generated. Each ALERT has the format
test-name_ALERT_alert-type_alert-level.
Click on the hyperlinks for more details of the test.

Alert level C

```
PLAT230_ALERT_2_C Hirshfeld Test Diff for    S1       --O1       .         5.8 s.u.
PLAT230_ALERT_2_C Hirshfeld Test Diff for    S1       --O2       .         6.3 s.u.
PLAT911_ALERT_3_C Missing FCF Refl Between Thmin & STh/L=    0.600          18 Report
```

Alert level G

```
ABSMU01_ALERT_1_G  Calculation of _exptl_absorpt_correction_mu
                not performed for this radiation type.
PLAT007_ALERT_5_G Number of Unrefined Donor-H Atoms ..............           1 Report
PLAT605_ALERT_4_G Largest Solvent Accessible VOID in the Structure         138 A**3
PLAT793_ALERT_4_G Model has Chirality at C8          (Centro SPGR)           S Verify
PLAT869_ALERT_4_G ALERTS Related to the Use of SQUEEZE Suppressed            ! Info
PLAT883_ALERT_1_G No Info/Value for _atom_sites_solution_primary .     Please Do !
PLAT912_ALERT_4_G Missing # of FCF Reflections Above STh/L=  0.600         145 Note
PLAT913_ALERT_3_G Missing # of Very Strong Reflections in FCF ....           2 Note
PLAT933_ALERT_2_G Number of OMIT Records in Embedded .res File ...           6 Note
PLAT941_ALERT_3_G Average HKL Measurement Multiplicity ...........         4.4 Low
PLAT978_ALERT_2_G Number C-C Bonds with Positive Residual Density.           5 Info
```

---

0 **ALERT level A** = Most likely a serious problem - resolve or explain
0 **ALERT level B** = A potentially serious problem, consider carefully
3 **ALERT level C** = Check. Ensure it is not caused by an omission or oversight
11 **ALERT level G** = General information/check it is not something unexpected

2 ALERT type 1 CIF construction/syntax error, inconsistent or missing data
4 ALERT type 2 Indicator that the structure model may be wrong or deficient
3 ALERT type 3 Indicator that the structure quality may be low
4 ALERT type 4 Improvement, methodology, query or suggestion
1 ALERT type 5 Informative message, check

It is advisable to attempt to resolve as many as possible of the alerts in all categories. Often the minor alerts point to easily fixed oversights, errors and omissions in your CIF or refinement strategy, so attention to these fine details can be worthwhile. In order to resolve some of the more serious problems it may be necessary to carry out additional measurements or structure refinements. However, the purpose of your study may justify the reported deviations and the more serious of these should normally be commented upon in the discussion or experimental section of a paper or in the "special_details" fields of the CIF. checkCIF was carefully designed to identify outliers and unusual parameters, but every test has its limitations and alerts that are not important in a particular case may appear. Conversely, the absence of alerts does not guarantee there are no aspects of the results needing attention. It is up to the individual to critically assess their own results and, if necessary, seek expert advice.

Publication of your CIF in IUCr journals

A basic structural check has been run on your CIF. These basic checks will be run on all CIFs submitted for publication in IUCr journals (*Acta Crystallographica*, *Journal of Applied Crystallography*, *Journal of Synchrotron Radiation*); however, if you intend to submit to *Acta Crystallographica Section C* or *E* or *IUCrData*, you should make sure that full publication checks are run on the final version of your CIF prior to submission.

Publication of your CIF in other journals

Please refer to the *Notes for Authors* of the relevant journal for any special instructions relating to CIF submission.

PLATON version of 13/07/2021; check.def file version of 13/07/2021

Datablock a_sq - ellipsoid plot

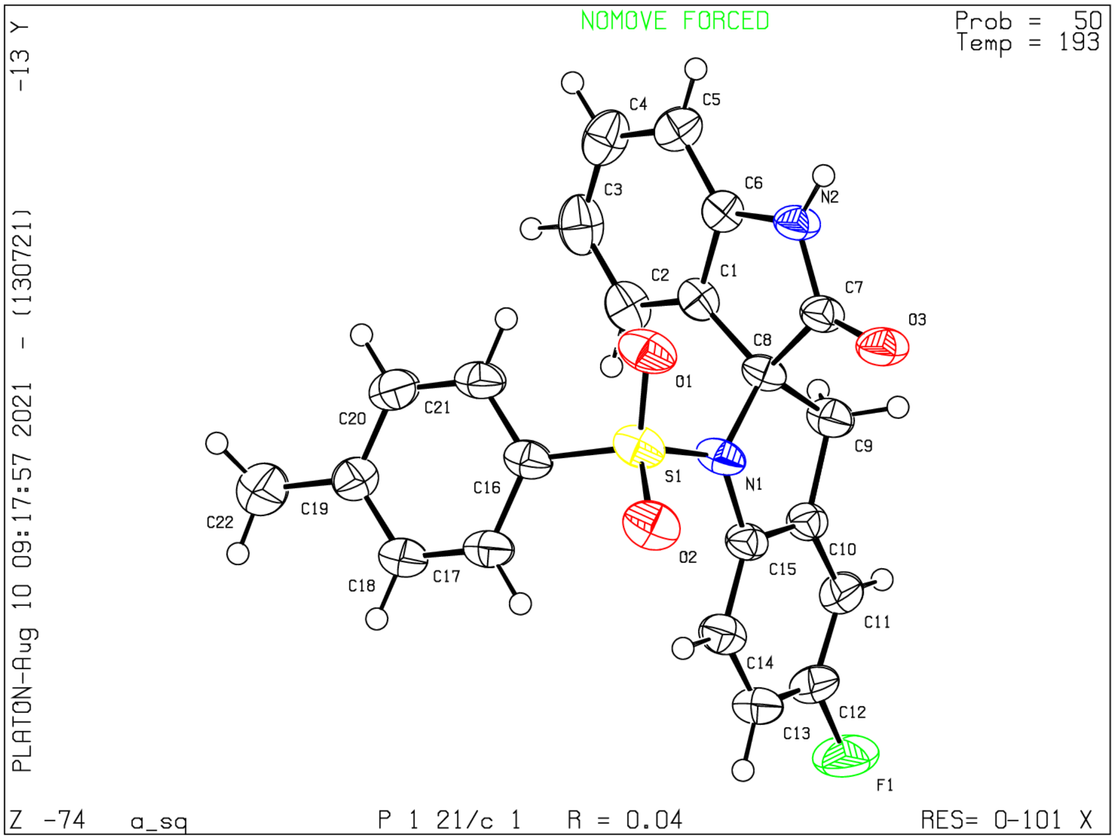

Supplement: Supplementary file 1 [file DataSheet2.PDF]
