# Supplementary material for: Efficient Synthesis of 2,3′-Spirobi (Indolin)-2′-Ones and Preliminary Evaluation of Their Damage to Mitochondria in HeLa Cells
Source: Front Pharmacol. 2022 Feb 23;12:821518. doi: 10.3389/fphar.2021.821518 (PMC8904893; doi:10.3389/fphar.2021.821518)
Supplement: Supplementary file 2 [file DataSheet1.PDF]

Supplementary Material

Efficient synthesis of 2,3'-spirobi[indolin]-2'-ones and preliminary evaluation of their damage to mitochondria in Hela Cells

Huajie Li[1, 2, †], Zhenjie Yu[1, 2, †], Haoyi Sun[1, 2, †], Bo Liu[1, 2], Xin Wang[1, 2], Zhe Shao[1, 2], Meiling Wang[1, 2], Weilin Xie[1, 2], Xingang Yao[3], Qingqiang Yao[1, 2*], Ying Zhi[1, 2*]

Table of Contents

1. NMR Spectra of 3a-3n --- S2

2. Antiproliferative activity ---S16

1. Copies of NMR Spectra

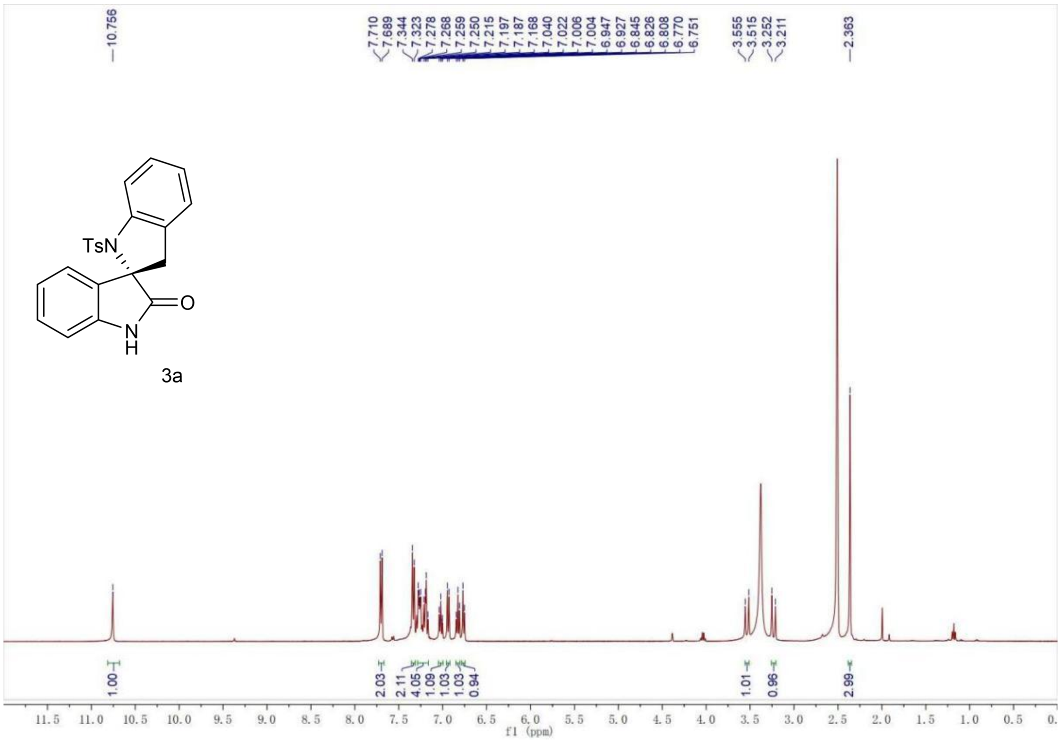

Supplementary Figure 1. ^{1}H NMR (400 MHz, DMSO-d_6) of **3a**

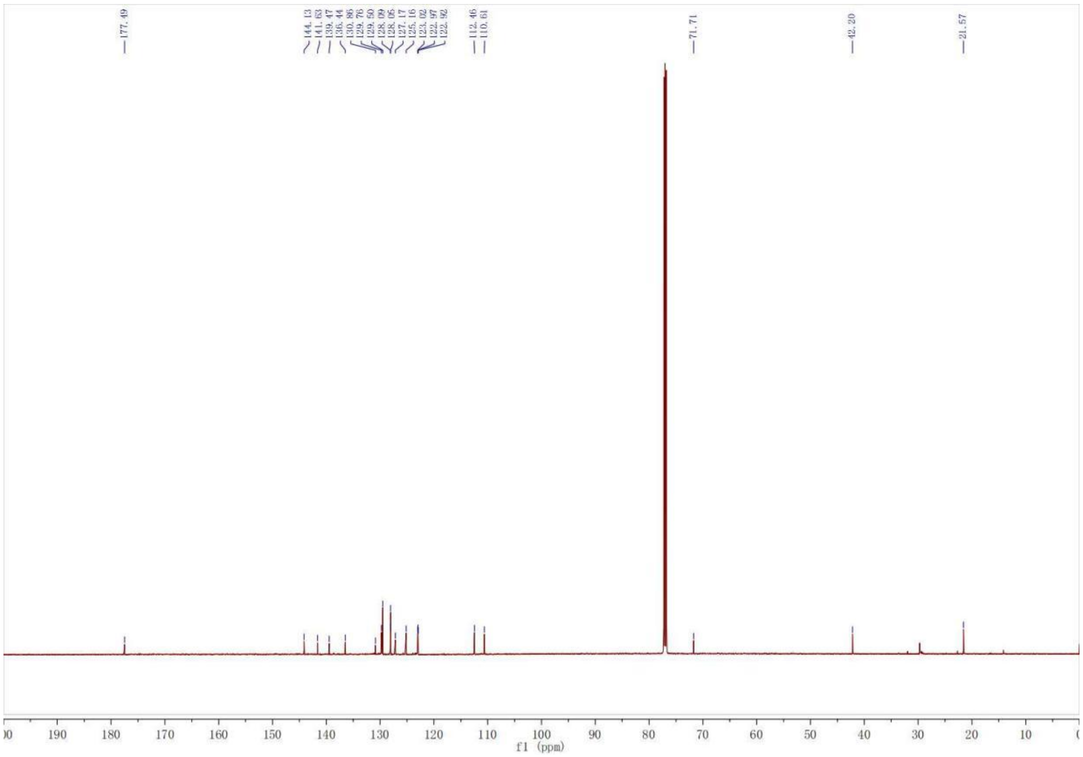

Supplementary Figure 2. ^{13}C NMR (150 MHz, $CDCl_3$) of **3a**

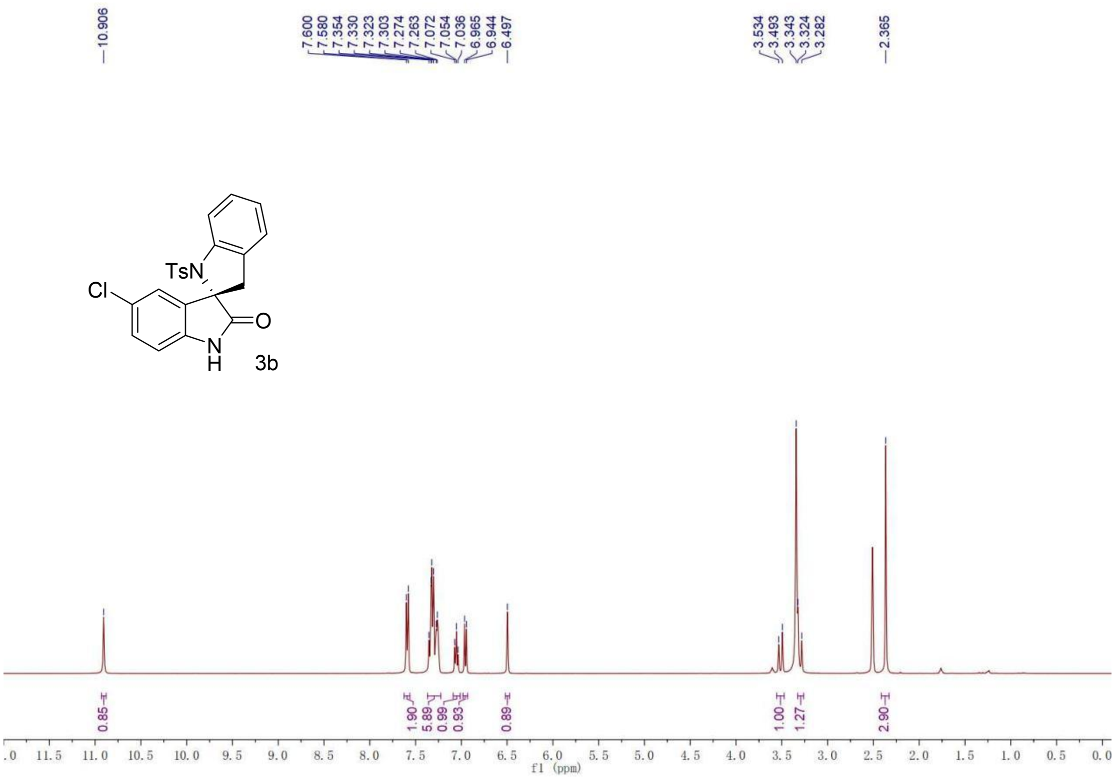

Supplementary Figure 3. ^{1}H NMR (400 MHz, DMSO-d_6) of **3b**

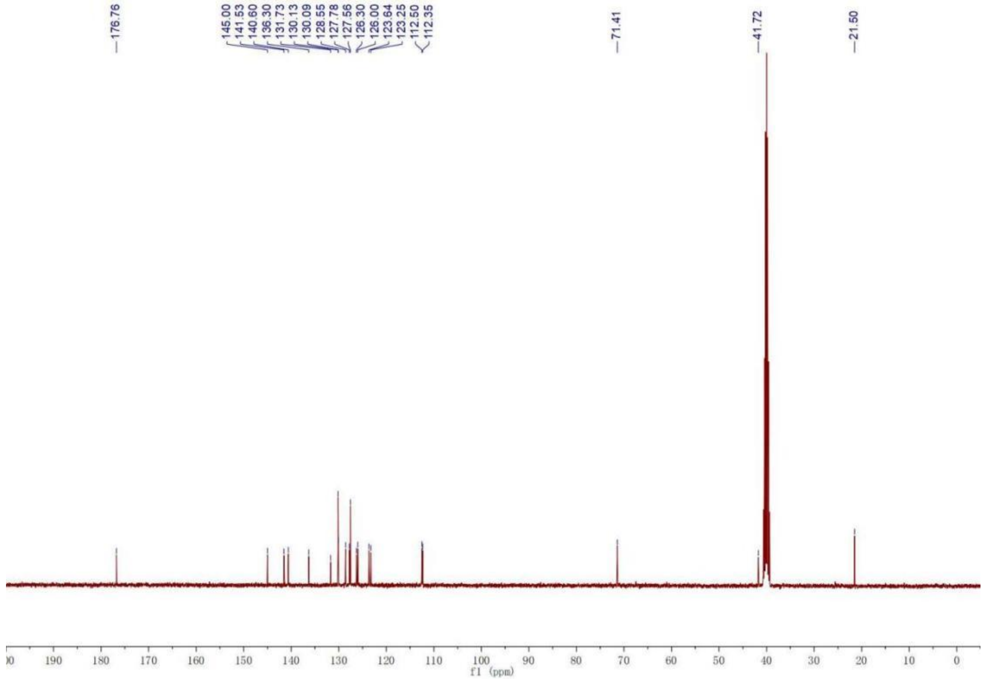

Supplementary Figure 4. ^{13}C NMR (100 MHz, DMSO-d_6) of **3b**

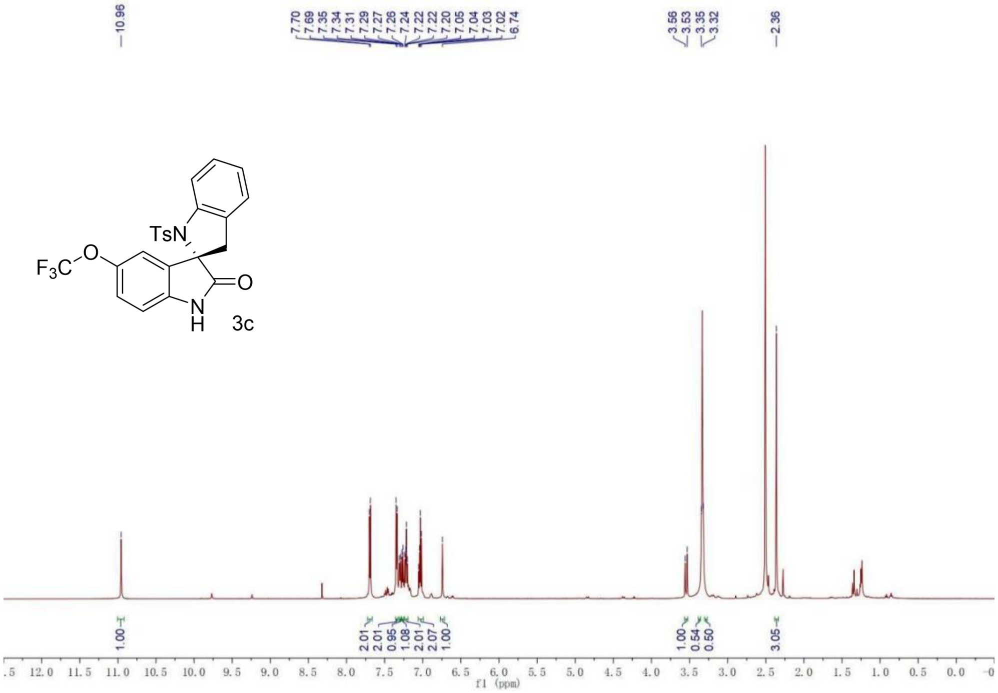

Supplementary Figure 5. ^{1}H NMR (400 MHz, DMSO-d_6) of **3c**

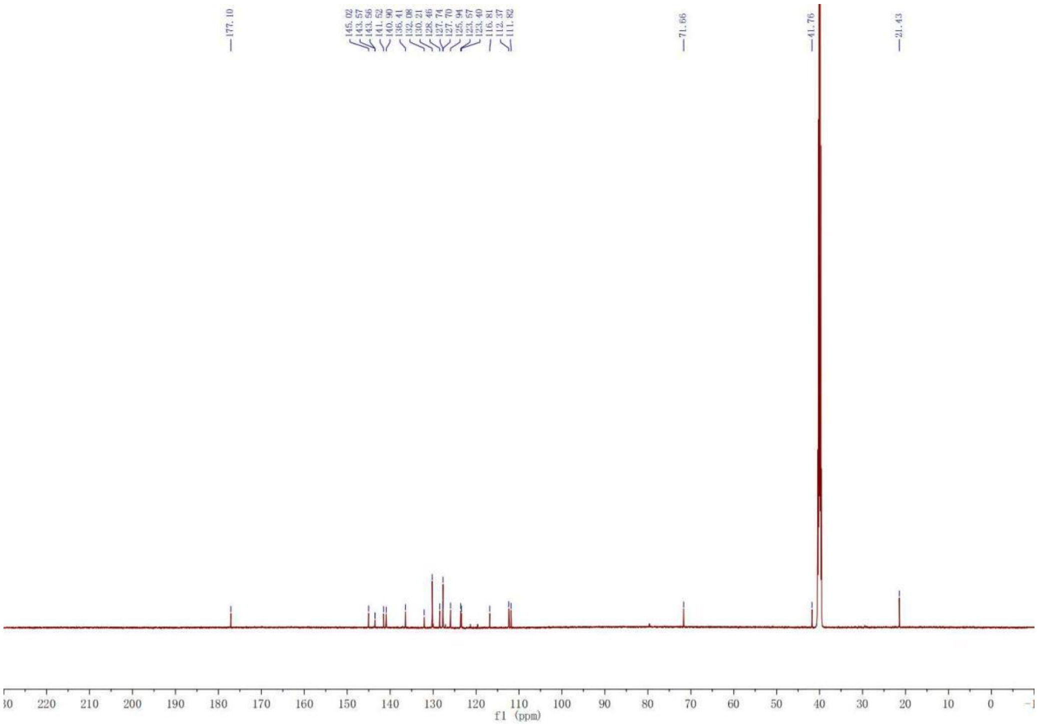

Supplementary Figure 6. ^{13}C NMR (100 MHz, DMSO-d_6) of **3c**

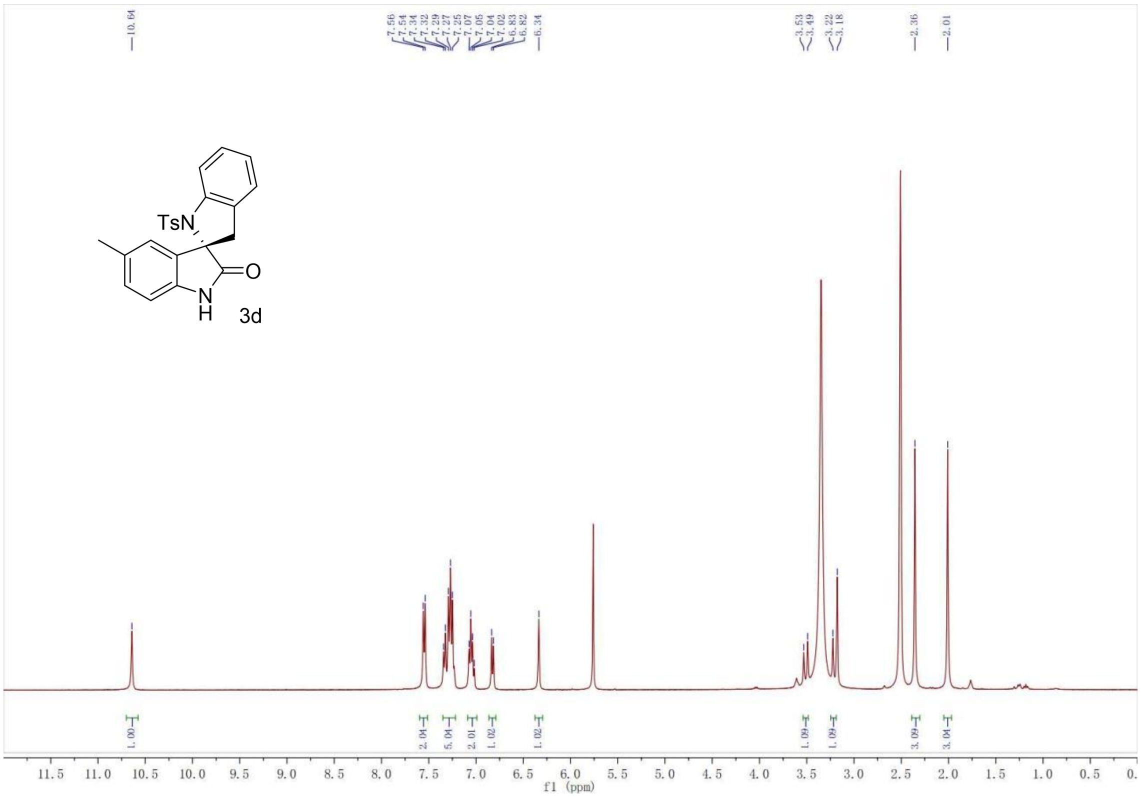

Supplementary Figure 7. ^{1}H NMR (600 MHz, DMSO-d_6) of **3d**

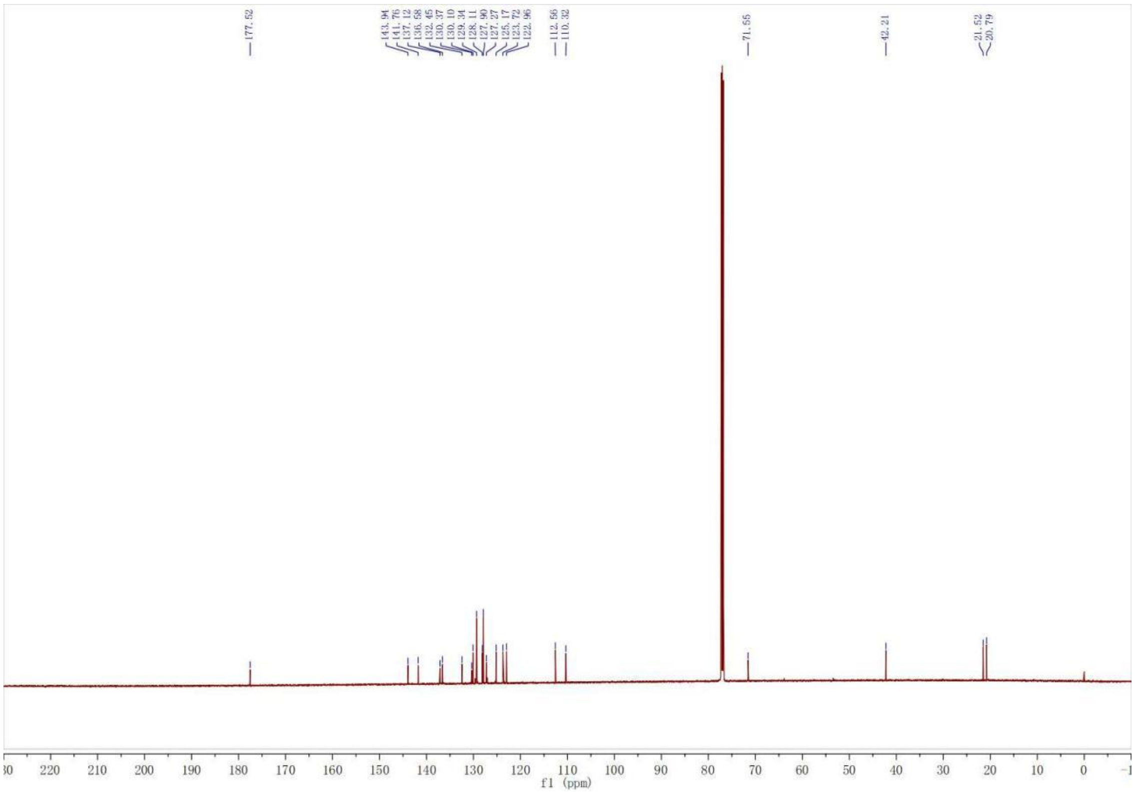

Supplementary Figure 8. ^{13}C NMR (150 MHz, DMSO-d_6) of **3d**

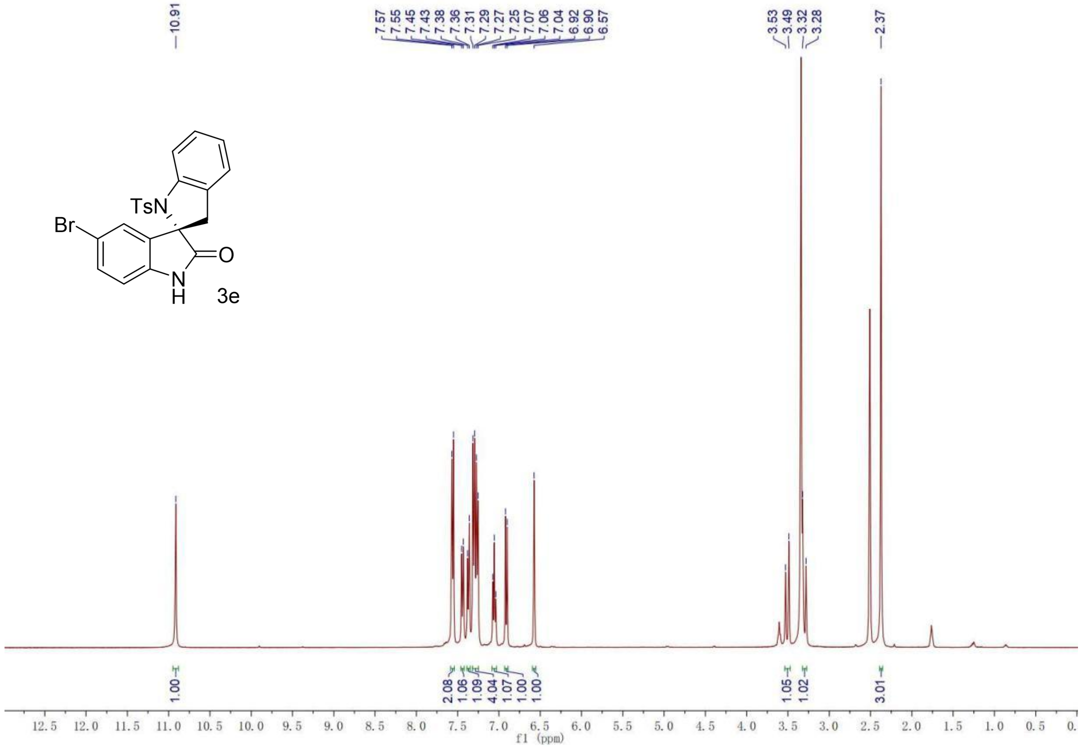

Supplementary Figure 9. ^{1}H NMR (400 MHz, DMSO-d_6) of **3e**

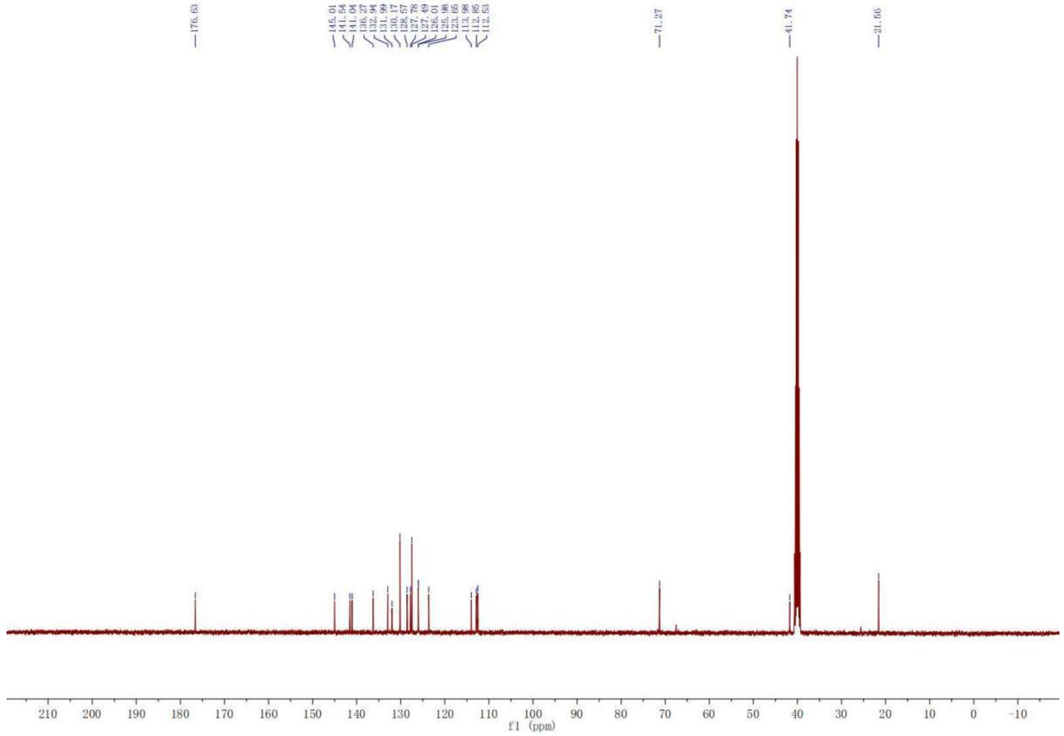

Supplementary Figure 10. ^{13}C NMR (125 MHz, $CDCl_3$) of **3e**

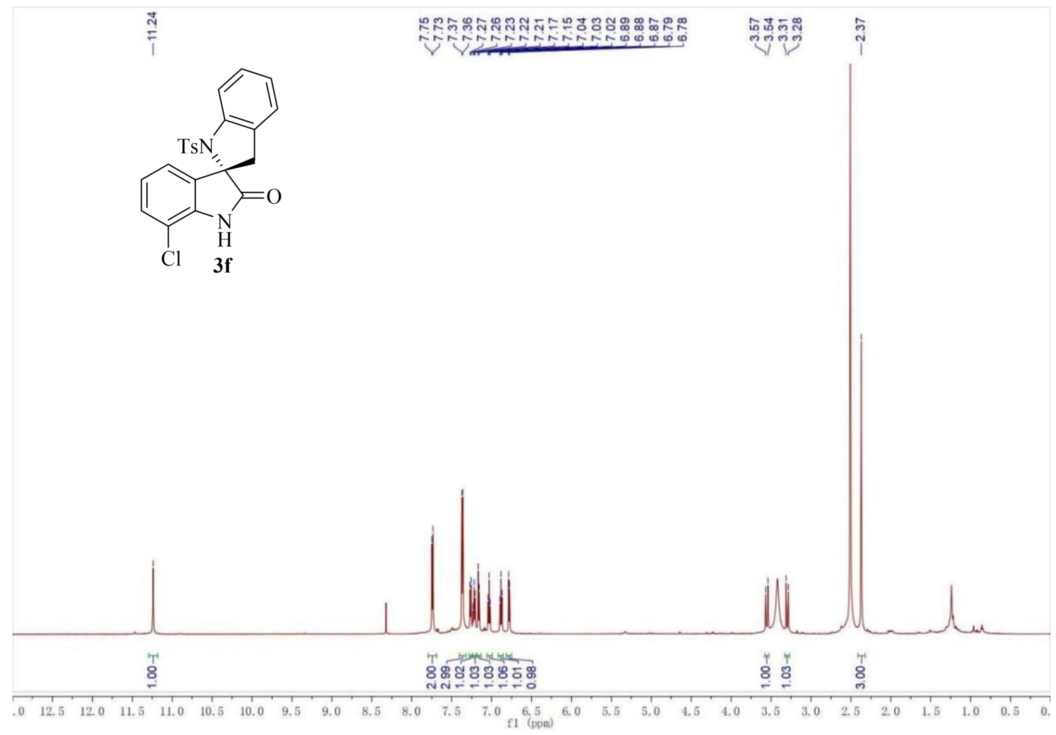

Supplementary Figure 11. ^{1}H NMR (400 MHz, DMSO-d_6) of **3f**

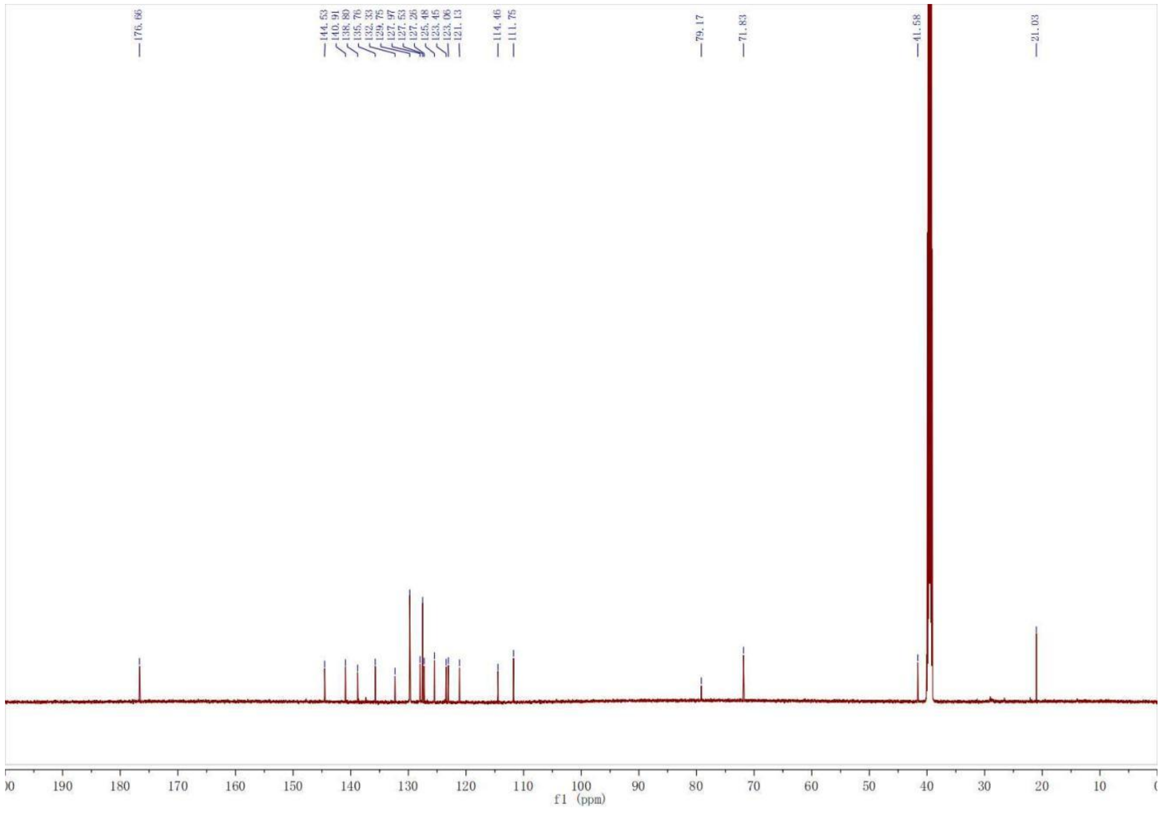

Supplementary Figure 12. ^{13}C NMR (100 MHz, DMSO-d_6) of **3f**

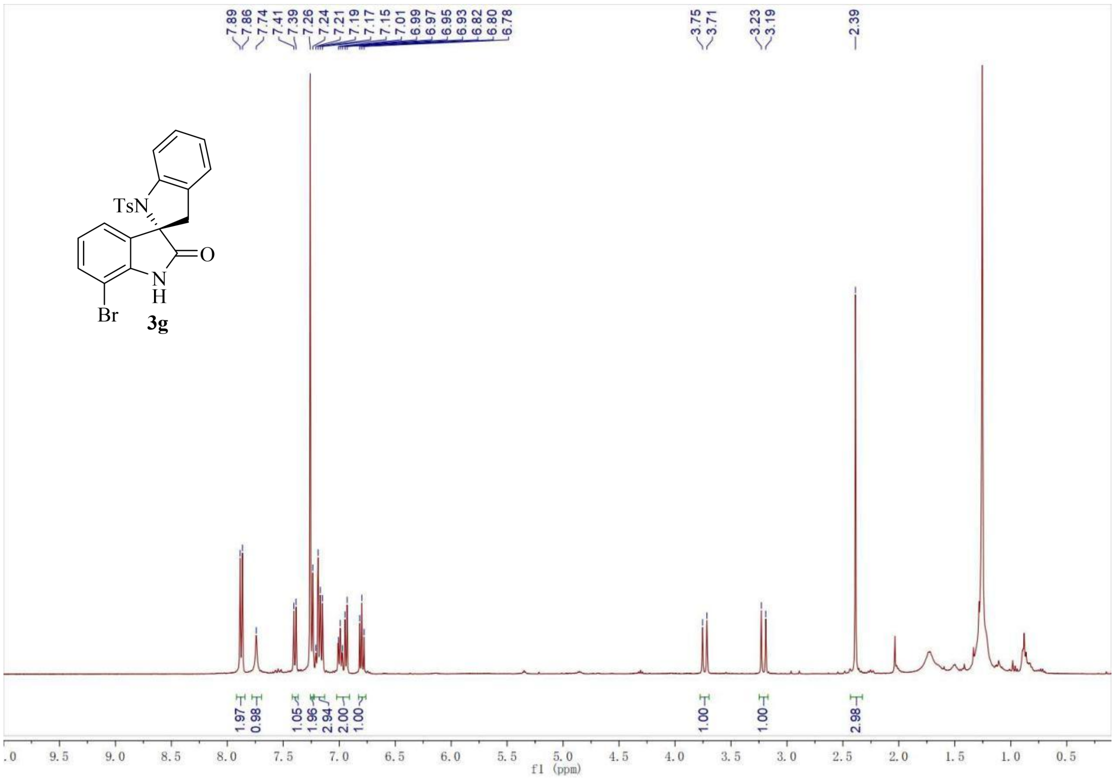

Supplementary Figure 13. ^{1}H NMR (600 MHz, $CDCl_3$) of **3g**

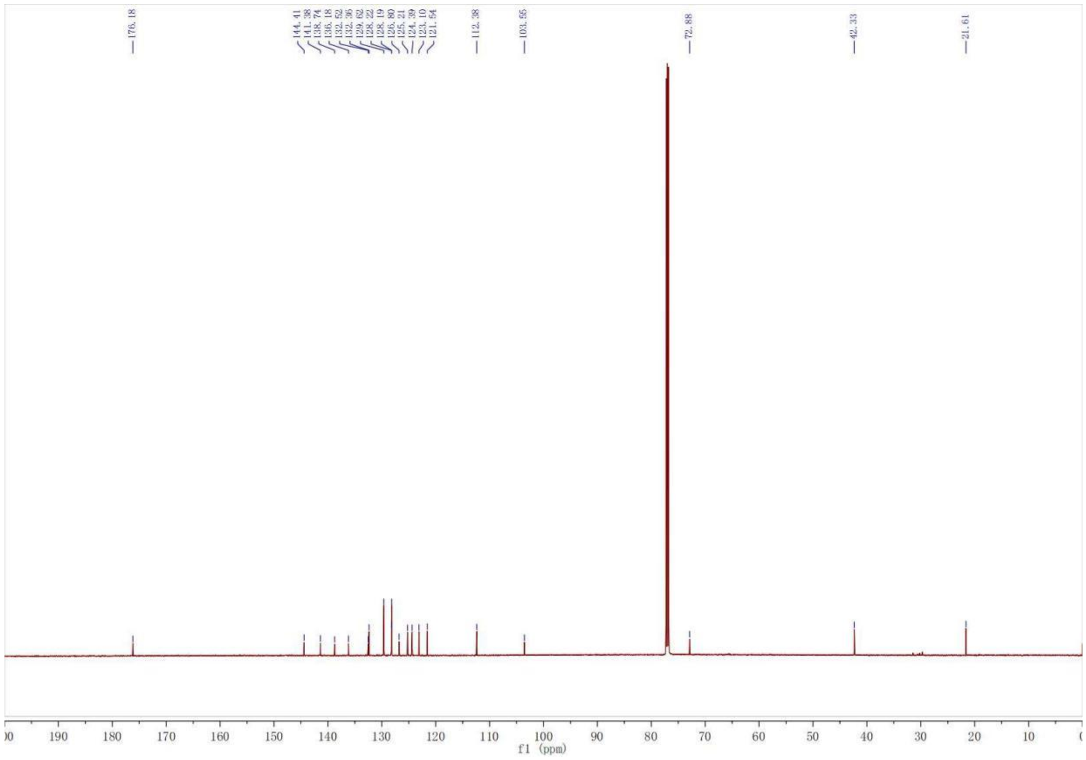

Supplementary Figure 14. ^{13}C NMR (150 MHz, $CDCl_3$) of **3g**

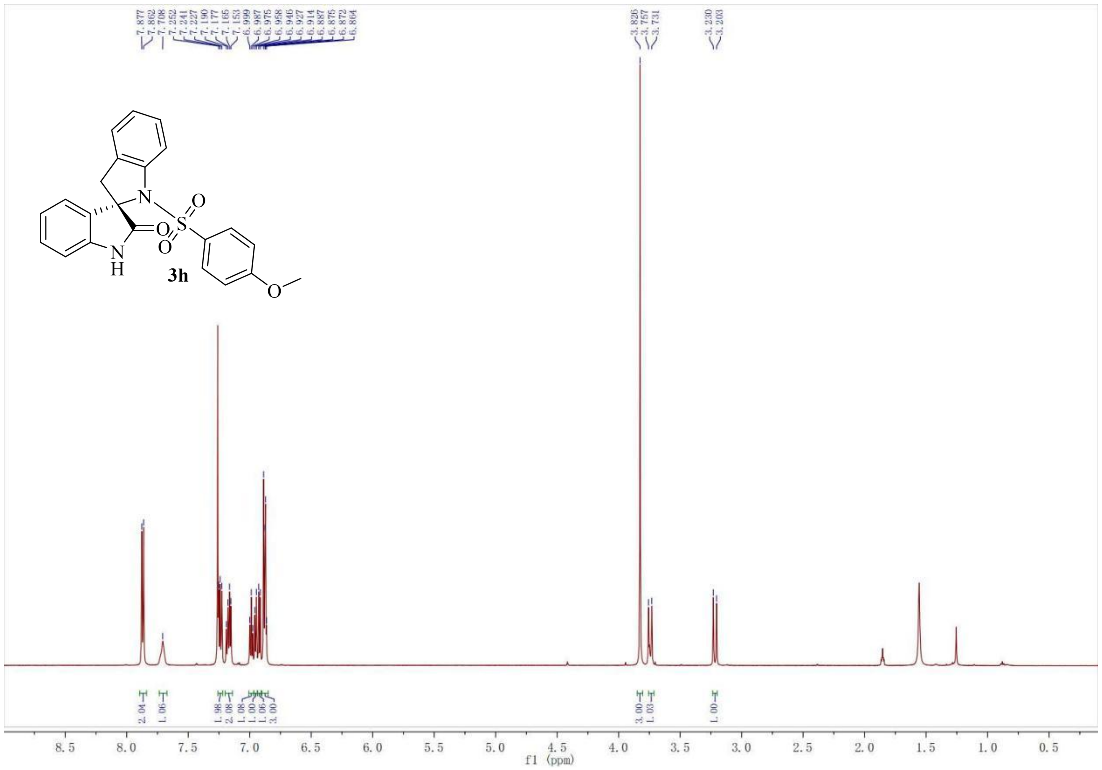

Supplementary Figure 15. ^{1}H NMR (400 MHz, $CDCl_3$) of **3h**

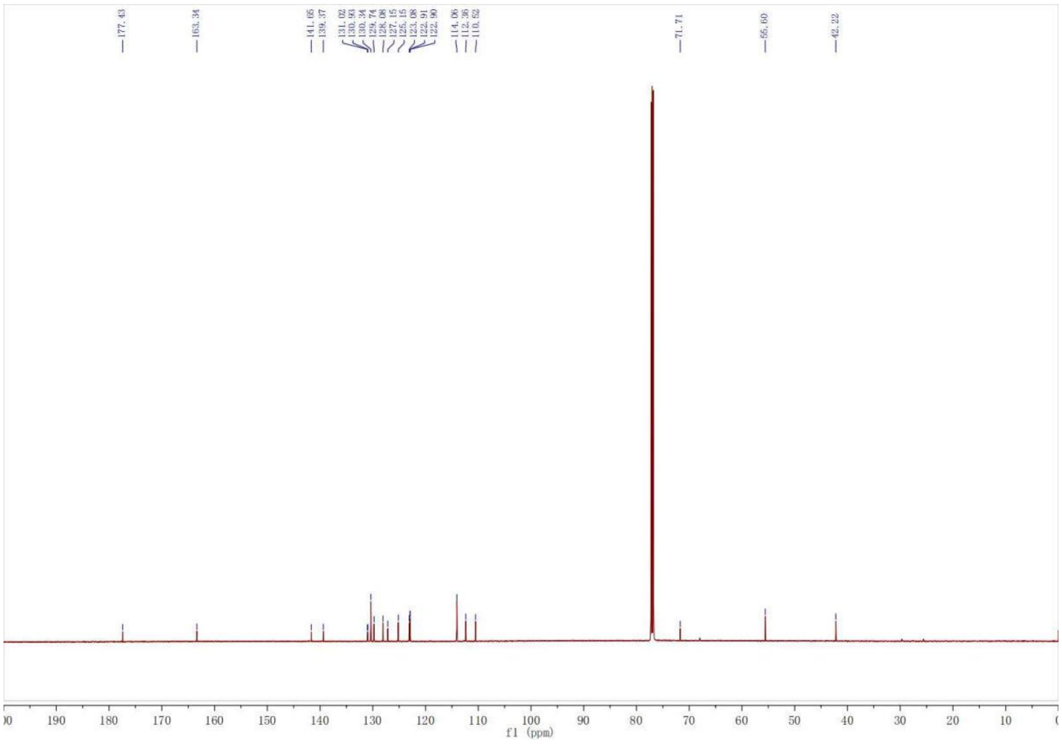

Supplementary Figure 16. ^{13}C NMR (150 MHz, $CDCl_3$) of **3h**

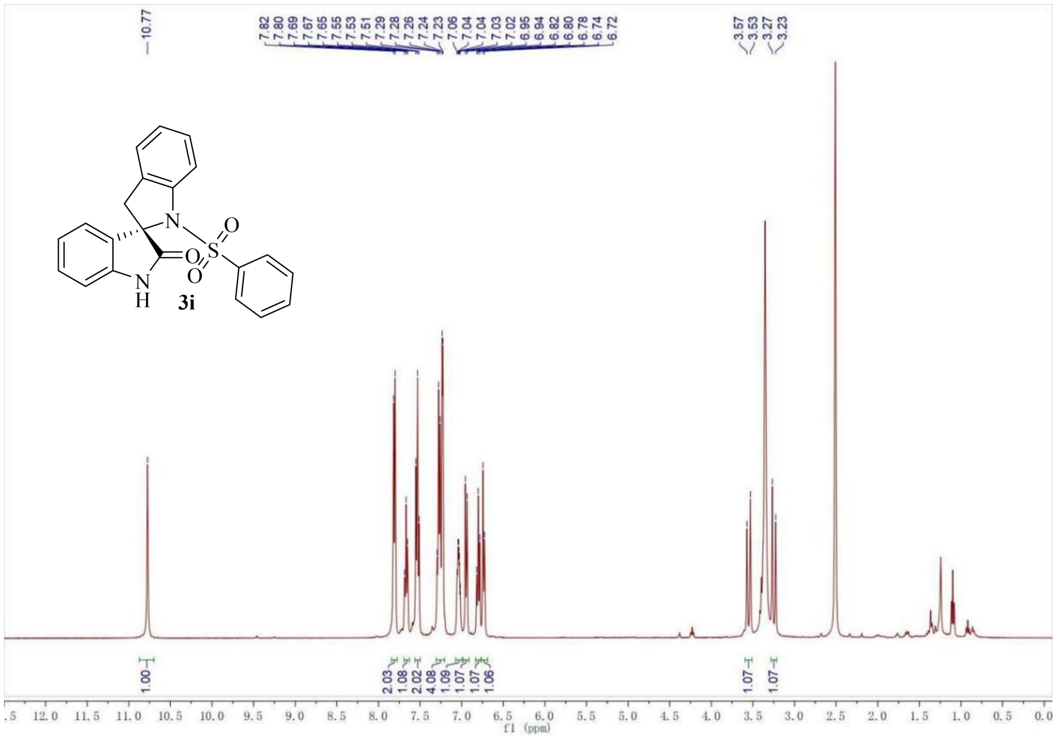

Supplementary Figure 17. ^{1}H NMR (600 MHz, DMSO-d_6) of **3i**

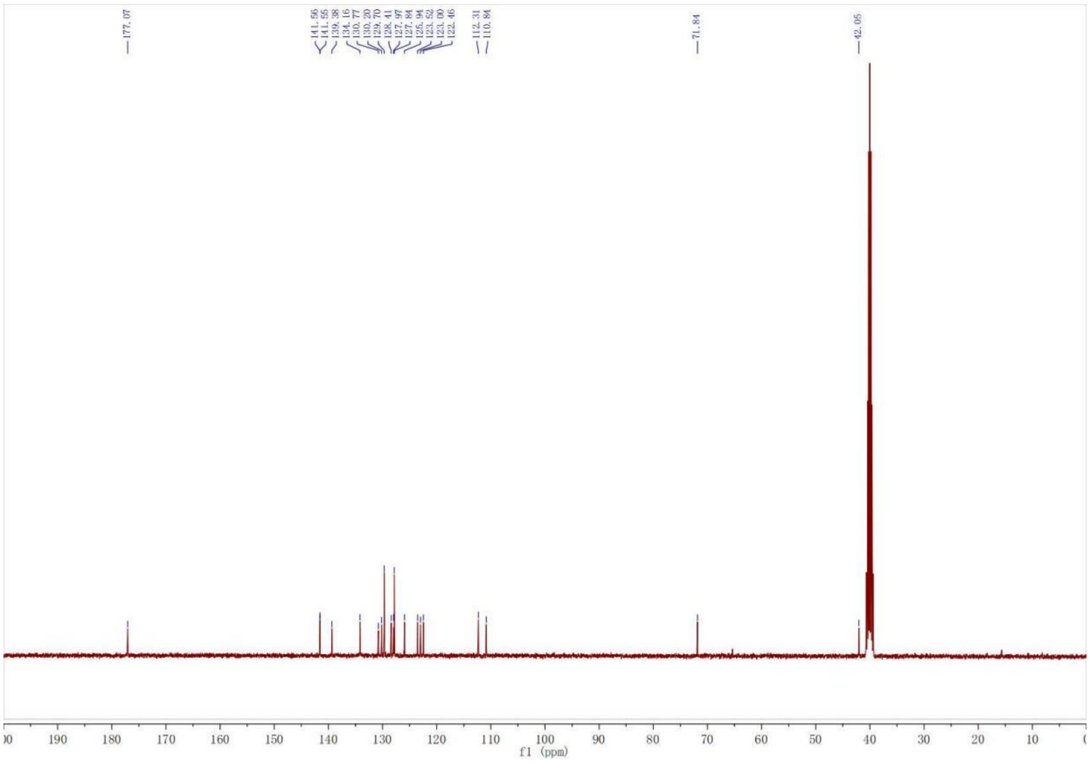

Supplementary Figure 18. ^{13}C NMR (150 MHz, DMSO-d_6) of **3i**

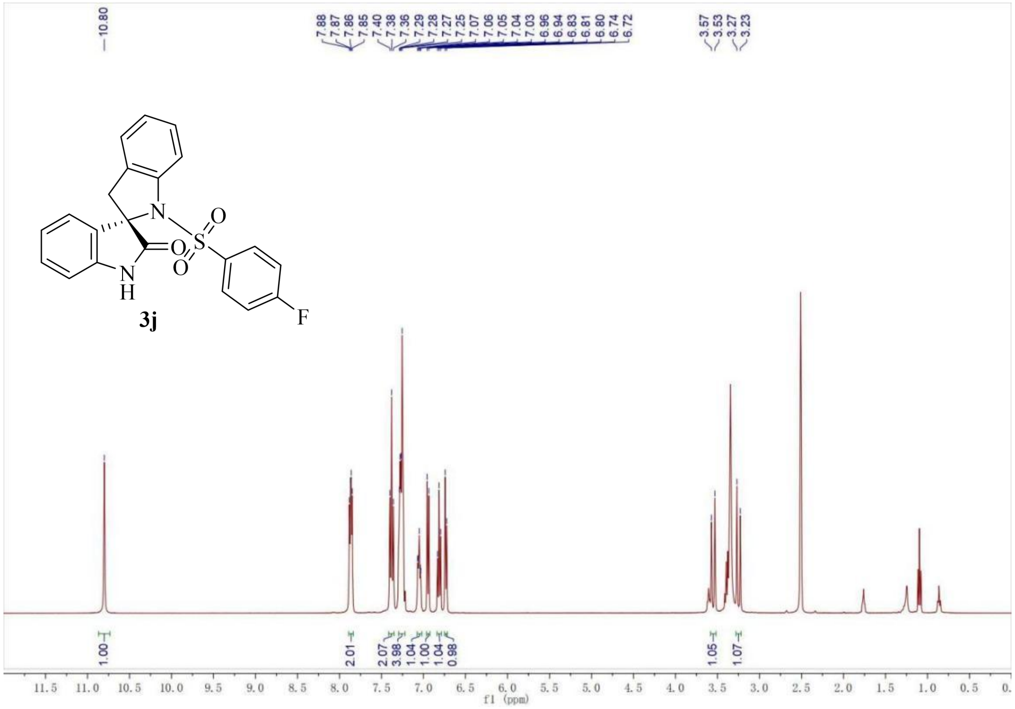

Supplementary Figure 19. ^{1}H NMR (400 MHz, DMSO-d_6) of **3j**

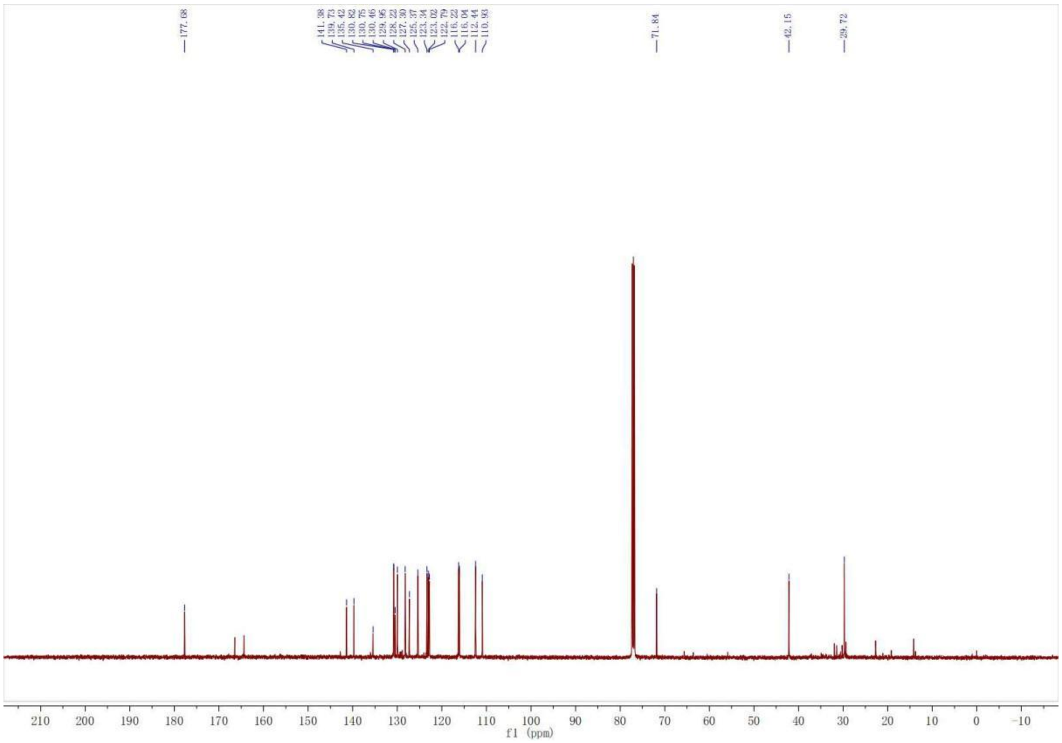

Supplementary Figure 20. ^{13}C NMR (100 MHz, DMSO-d_6) of **3j**

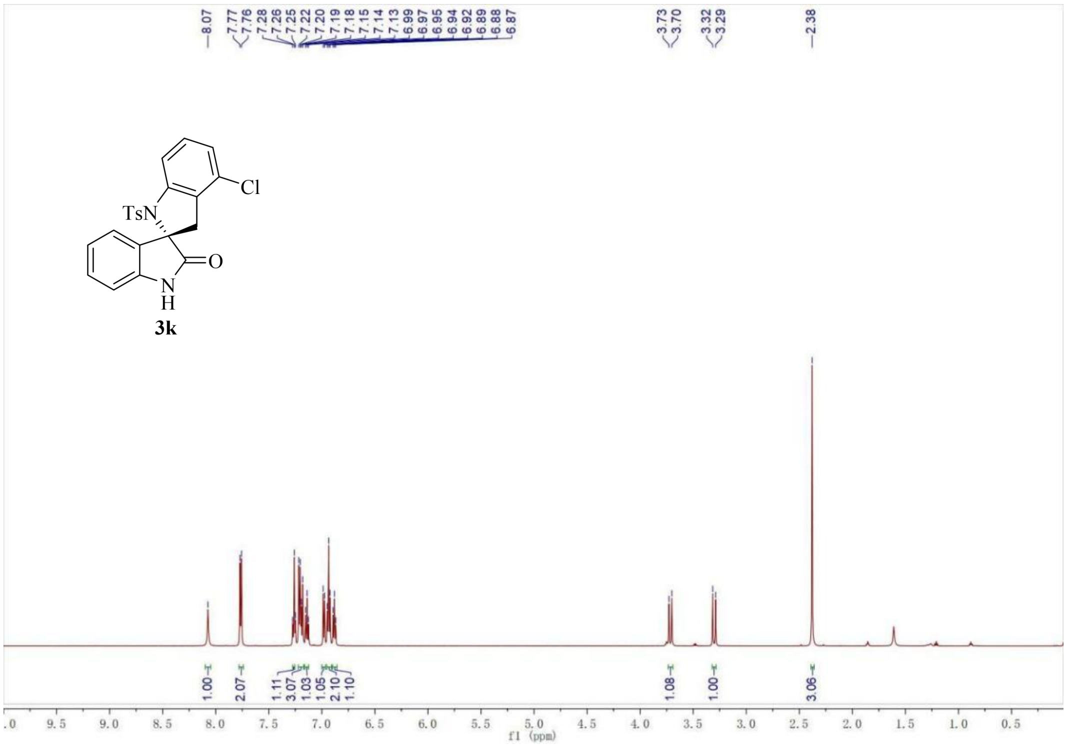

Supplementary Figure 21. ^{1}H NMR (600 MHz, $CDCl_3$) of **3k**

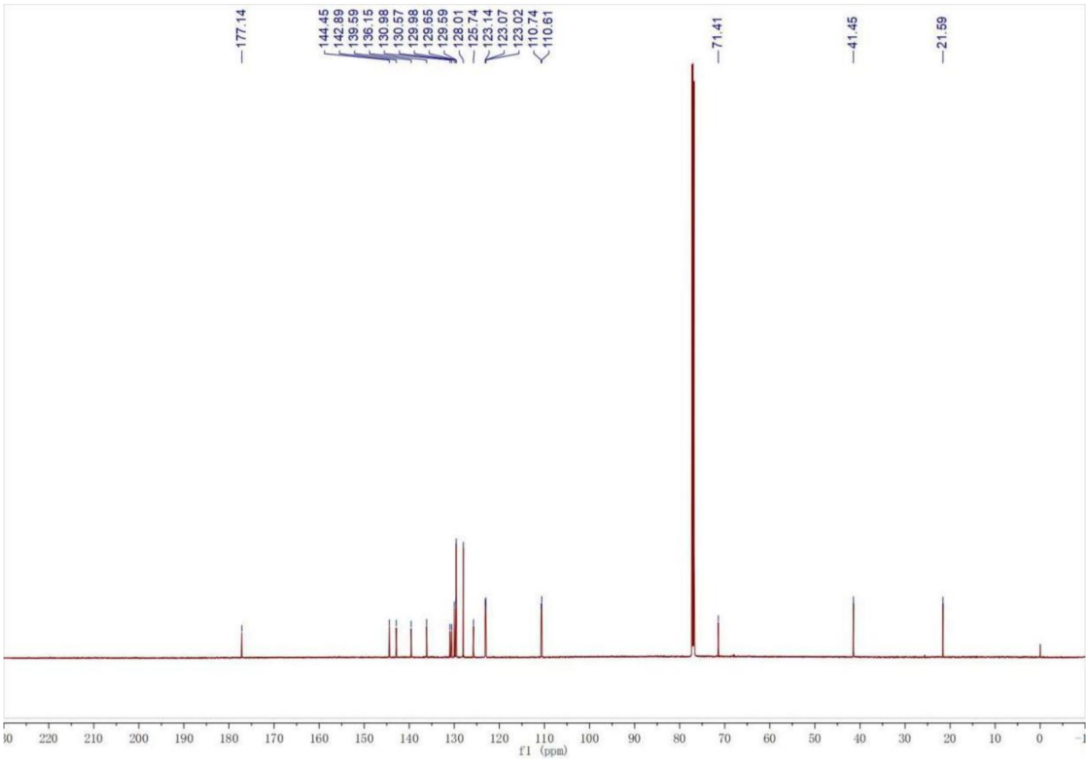

Supplementary Figure 22. ^{13}C NMR (150 MHz, DMSO-d_6) of **3k**

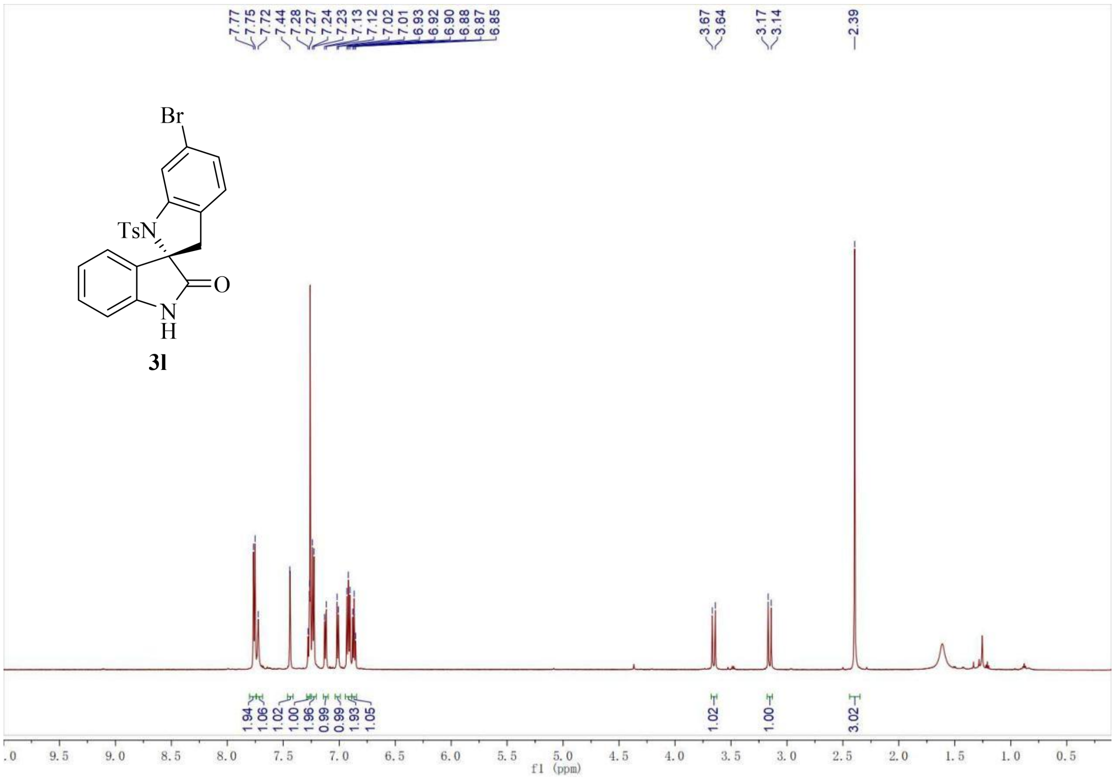

Supplementary Figure 23. ^{1}H NMR (600 MHz, $CDCl_3$) of **3l**

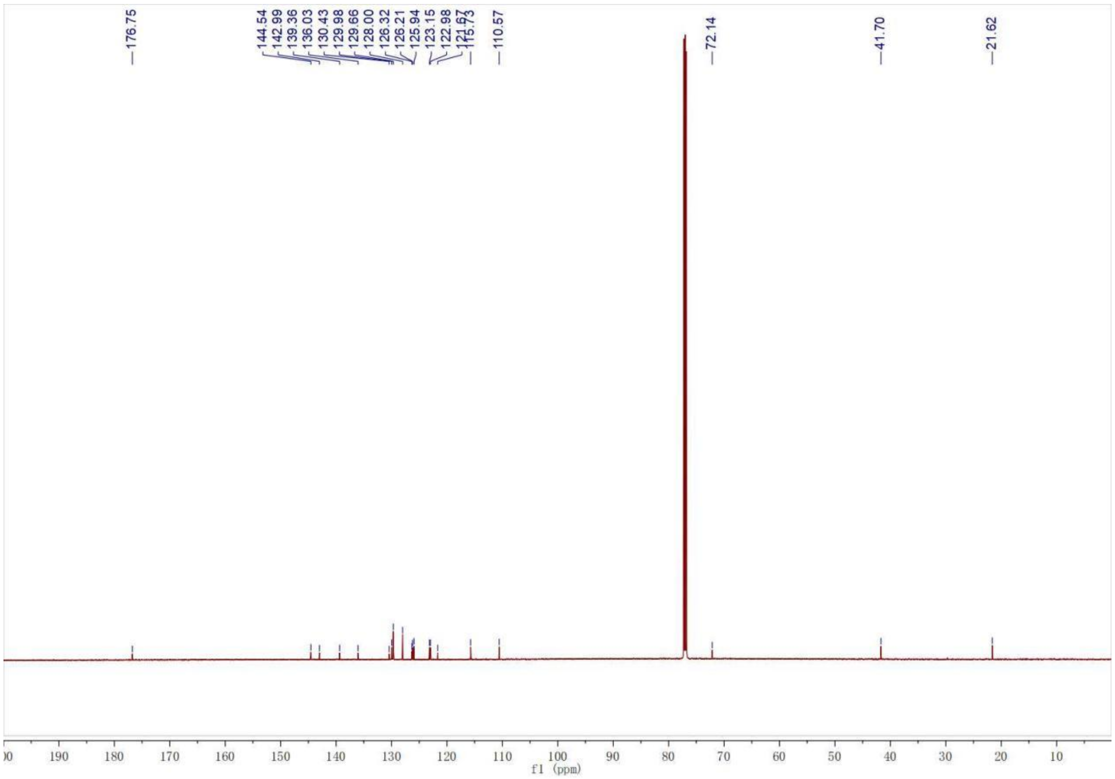

Supplementary Figure 24. ^{13}C NMR (150 MHz, $CDCl_3$) of **3l**

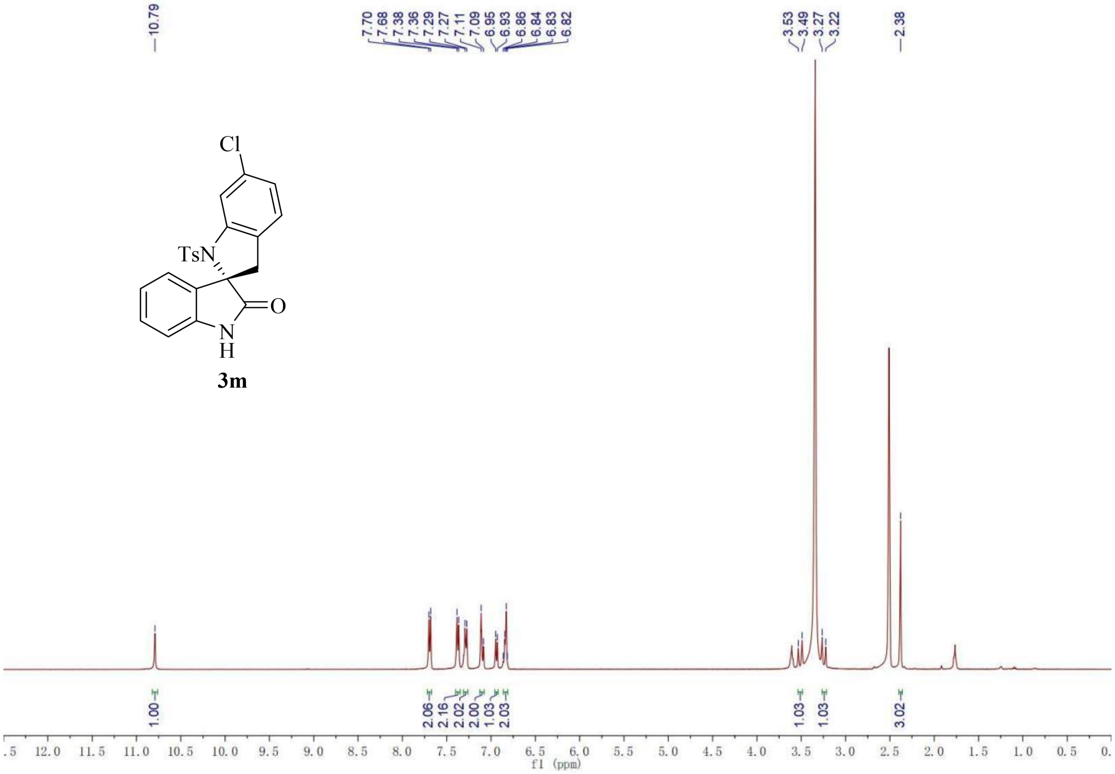

Supplementary Figure 25. ^{1}H NMR (400 MHz, DMSO-d_6) of **3m**

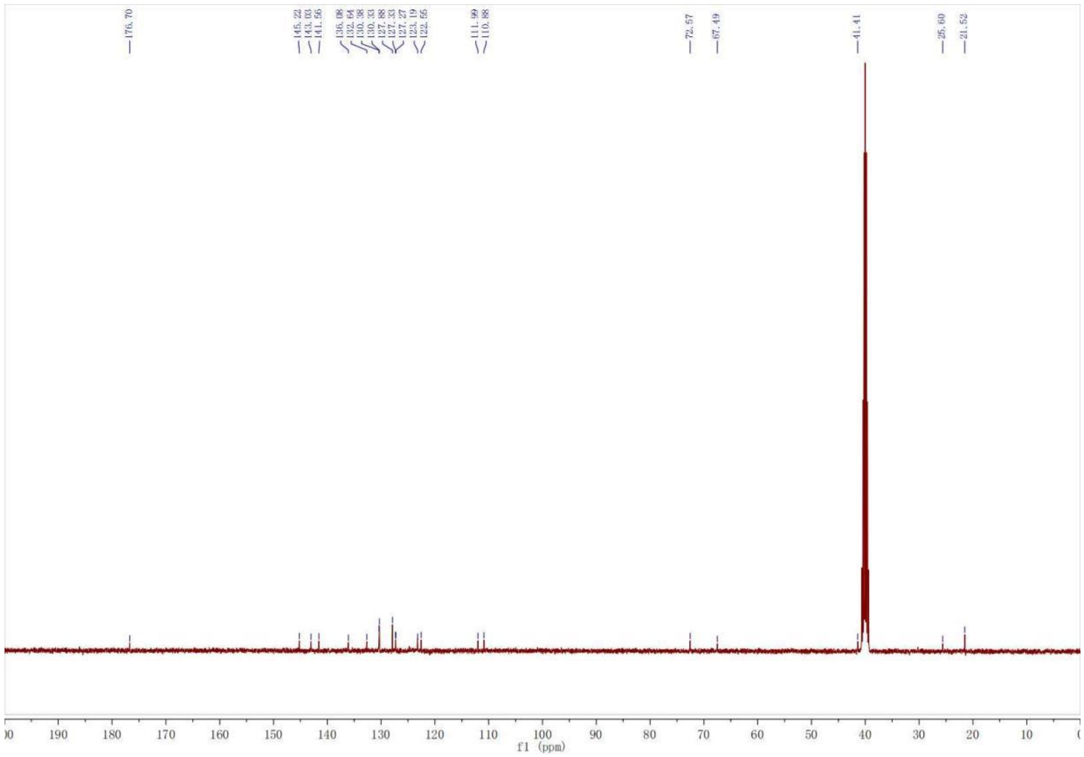

Supplementary Figure 26. ^{13}C NMR (100 MHz, DMSO-d_6) of **3m**

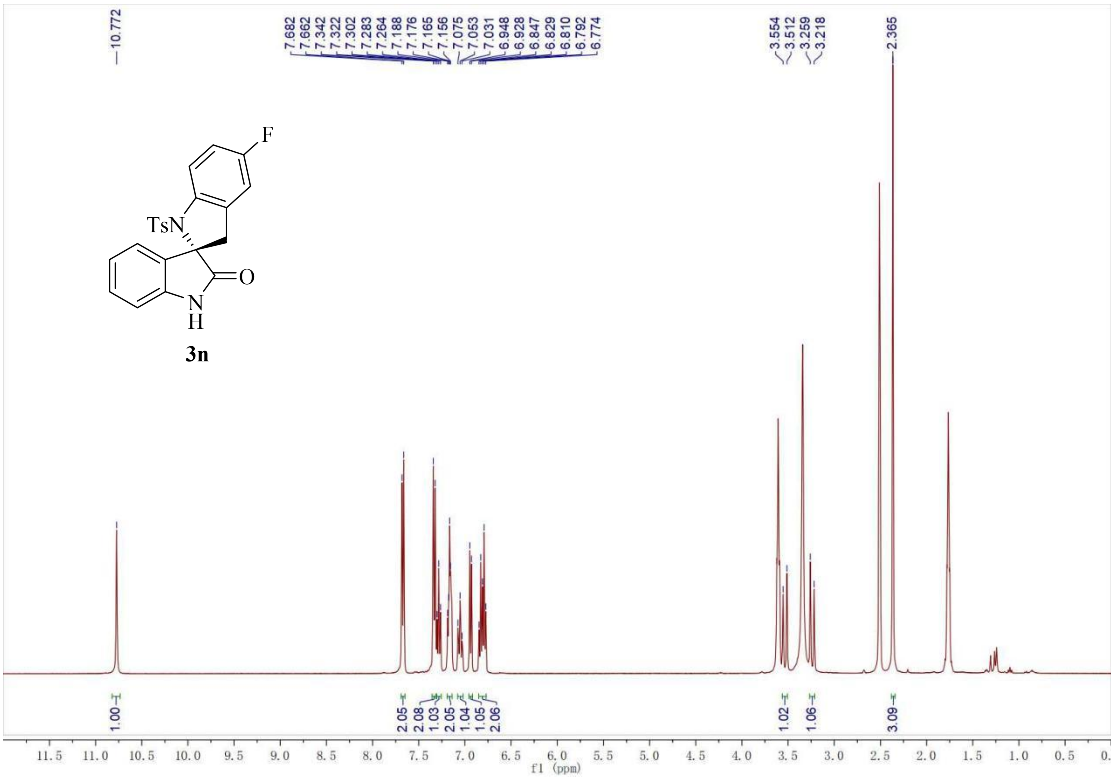

Supplementary Figure 27. ^{1}H NMR (400 MHz, DMSO-d_6) of **3n**

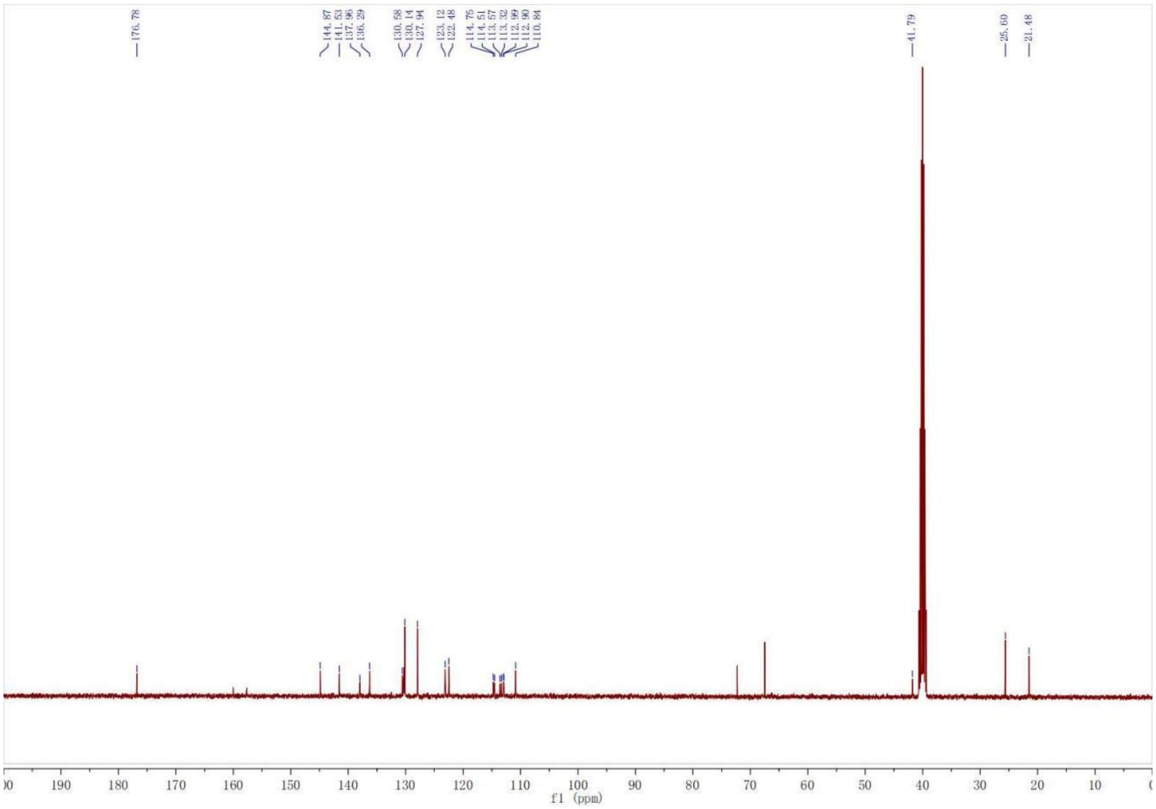

Supplementary Figure 28. ^{13}C NMR (150 MHz, $CDCl_3$) of **3n**

2. Antiproliferative activity

***3f (LHJ-129)** and **3g (LHJ-128)** exhibit antiproliferative activity against acute myeloid leukemia cell line HL60.*

To more broadly assess their bioactivity, we performed *antiproliferative activity test* in HL60 cells treated with 20 μM of a series of 2,3'-spirobi[indolin]-2'-one synthesized (Figure 29). Among all tested compounds, **3f** and **3g** displayed promising antiproliferative activity with inhibition ratio >50% at 25 μM. The final IC_{50} of **3f** and **3g** measured by MTT was 24.16 μM and 16.03 μM respectively suggesting that these compounds could be further developed as potential anti-tumor agents.

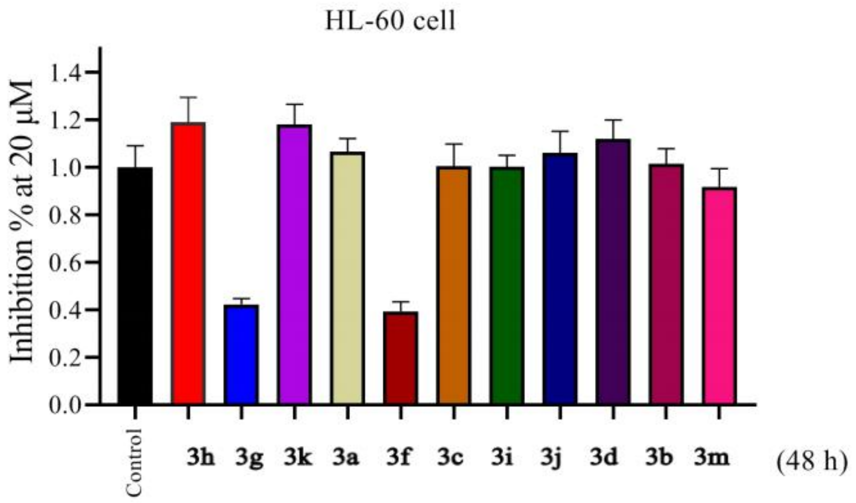

Supplementary Figure 29. Antiproliferative activity of representative compounds against acute myeloid leukemia cell line HL60. Data represents the mean inhibition ratio of proliferation at 20 μM, error bar represents SD value, three experiments were performed for each data.

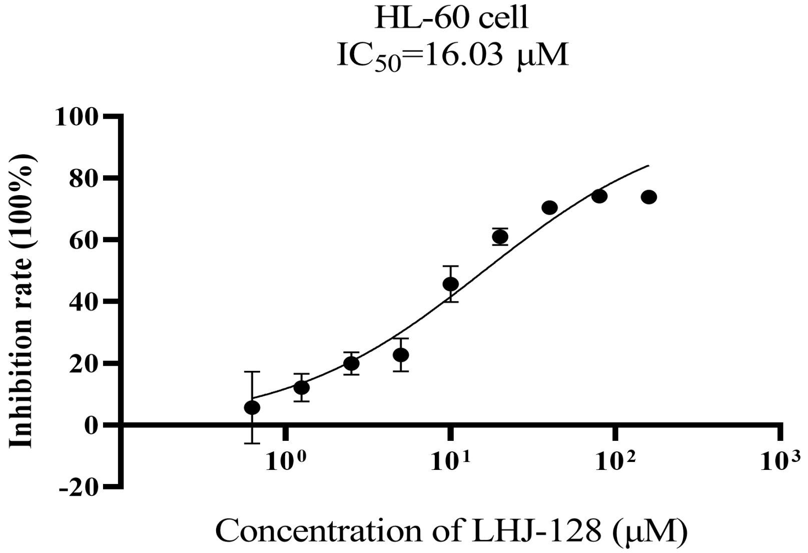

Supplementary Figure 30. Antiproliferative activity of **3g** against acute myeloid leukemia cell line HL60

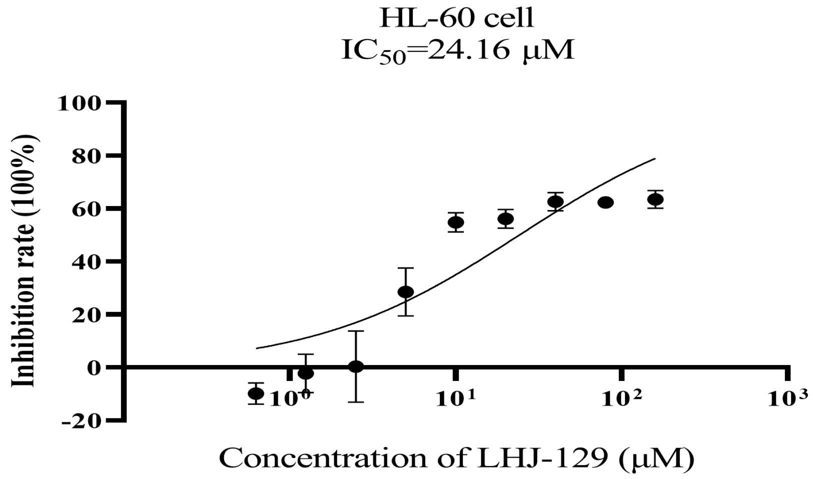

Supplementary Figure 31. Antiproliferative activity of **3f** against acute myeloid leukemia cell line HL60

Method and Materials

HL-60 cells was plated into the 96-well cell plate at $1x10^4$ cell/ml overnight, then small molecule compounds, **3g** and **3f**, were added into the cell medium for another 20h at the indicated concentration respectively. MTT (5 mg/ml) was added into the cell medium at 10 μL/well for 4h. After discarding the culture solution after centrifugation, the culture was terminated and prepared to dissolve the crystals with DMSO (150 μL). After all the crystals were dissolved, the absorbance at 570nm was analyzed with spectrophotometer.
